# Looping specificity of Polycomb response elements requires GAF and a combination of looping factors that could form a code

**DOI:** 10.1093/nar/gkag512

**Published:** 2026-05-28

**Authors:** Gonzalo Sabarís, Marco Di Stefano, Sandrine Denaud, Lauriane Fritsch, Ana-Maria Popmihaylova, Giorgio-Lucio Papadopoulos, Bernd Schuettengruber, Giacomo Cavalli

**Affiliations:** IGH, UMR9002, Univ Montpellier, CNRS, 141 Rue de la Cardonille, 34396, Montpellier Cedex 5, France; Institut Botànic de Barcelona (IBB), CSIC-CMCNB, Passeig del Migdia, s/n, Sants-Montjuïc, 08038, Barcelona, Catalonia, Spain; IGH, UMR9002, Univ Montpellier, CNRS, 141 Rue de la Cardonille, 34396, Montpellier Cedex 5, France; IGH, UMR9002, Univ Montpellier, CNRS, 141 Rue de la Cardonille, 34396, Montpellier Cedex 5, France; IGH, UMR9002, Univ Montpellier, CNRS, 141 Rue de la Cardonille, 34396, Montpellier Cedex 5, France; IGH, UMR9002, Univ Montpellier, CNRS, 141 Rue de la Cardonille, 34396, Montpellier Cedex 5, France; IGH, UMR9002, Univ Montpellier, CNRS, 141 Rue de la Cardonille, 34396, Montpellier Cedex 5, France; IGH, UMR9002, Univ Montpellier, CNRS, 141 Rue de la Cardonille, 34396, Montpellier Cedex 5, France; IGH, UMR9002, Univ Montpellier, CNRS, 141 Rue de la Cardonille, 34396, Montpellier Cedex 5, France

## Abstract

Chromatin looping between *cis*-regulatory elements is essential for precise developmental gene expression, and its disruption is frequently linked to disease. Polycomb response elements (PREs) are specialized tethering elements that mediate chromatin loops and are bound by transcription factors (TFs) like GAGA-associated factor (GAF), contributing to the recruitment of Polycomb group (PcG) proteins. While both PcG proteins and GAF have been implicated in looping, their specific roles and the mechanisms of loop specificity remain unresolved. Using genome-wide and locus-specific approaches, we show that high GAF occupancy is required for chromatin looping and gene regulation. However, GAF alone cannot establish loops without additional factors. Surprisingly, PRE looping does not require Polycomb repressive complex 1 (PRC1) or the repressive histone mark H3K27me3. Intriguingly, orthologous PRE sequences can rescue looping, while unrelated PREs with similar GAF levels cannot, indicating that looping specificity depends on both GAF levels and compatible factor combinations at loop anchors. Together, our results support a combinatorial model in which GAF collaborates with additional looping factors to drive PRE-specific interactions, suggesting that certain combinations of factors at PREs may determine which elements form loops and contribute to Polycomb-mediated gene silencing.

## Graphical Abstract

**Graphical Abstract:**
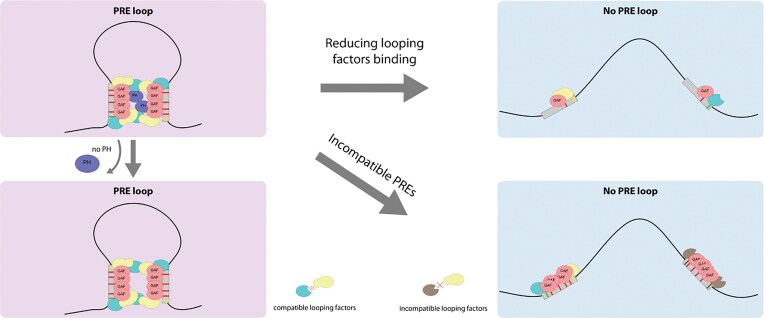

## Introduction

3D chromatin organization plays a critical role in genome function. In eukaryotes, interphase chromosomes are partitioned into physical domains (also defined as topologically associating domains or TADs), which promote local interactions between *cis*-regulatory elements (CREs) and insulate neighboring genomic regions [1–3]. Recently, high-resolution Micro-C experiments in early *Drosophila* embryos identified a class of *cis*-elements named “tethering elements” (TEs) that foster chromatin loops between CREs within TADs to prime genes for rapid activation [4].

Another class of CREs forming chromatin loops and involved in the maintenance of gene expression states of key developmental genes are Polycomb response elements (PREs) (reviewed in [5]). PREs act as nucleation sites for the recruitment of the Polycomb repressive complexes 2 and 1 (PRC2 and PRC1), which are responsible for the deposition and spreading of repressive histone marks (H3K27me3 and H2AK118ub, respectively) forming Polycomb domains [6]. These epigenetic domains often overlap with physical domains (TADs), although PcG-associated chromatin marks are dispensable for the formation of these TADs [7]. Most Polycomb domains contain multiple PREs and co-regulated genes, often involved in shared developmental pathways [8, 9]. Only a subset of PREs forms chromatin loops (PRE loops), which are established during early *Drosophila* embryogenesis, concurrent with zygotic genome activation [10–12]. PRE loops act as architectural scaffolds that form and persist independently of the underlying gene expression state [13, 14]. These loops can modulate gene expression by restricting enhancer–promoter communications or contributing to enhancer specificity [13]. Intriguingly, a large part of the PREs correspond to TEs, with which they share common features: both elements are bound by PcG proteins, such as the PRC1 subunit Polyhomeotic (PH), and by the GAGA factor (GAF), but not by typical insulator factors such as CTCF and CP190 [4]. This raises the questions of how such chromatin loops are formed and what determines their specificity, questions of increasing interest in the field.

GAF has emerged as a key player in looping formation and it is widely associated with TEs throughout the *Drosophila* genome [4, 15]. Similarly, it is highly enriched at PREs [12, 14] and loop anchors of co-expressed genes [16]. GAF possesses a BR-C, ttk and bab (BTB), or Pox virus and Zinc finger (POZ) oligomerization domain [17–19], known to mediate long-range DNA interactions [17, 20]. Deletion of the POZ domain results in the loss of a subset of chromatin loops, particularly between paralogous genes [15], suggesting that this domain is involved in chromatin looping between GAF bound sites. In addition, GAF is enriched at chromatin loops and TAD boundaries structuring the genome prior to zygotic genome activation in the early *Drosophila* embryo [21, 22]. Moreover, GAF is required for the formation of a subset of the so-called, meta-loops, chromatin contacts established at the multimegabase scale in the central nervous system [23]. Similarly, GAF can mediate long-range contacts between homologous PREs located on different chromosomes [24], and loss of GAF binding to the *dac* PRE results in reduced chromatin looping [12].

Despite this compelling evidence implicating GAF in chromatin looping, its specific function remains unclear. Only a small minority of all genomic GAF binding sites participate in loop formation, and genome-wide depletion of GAF in *Drosophila* embryos results in the loss of only a small fraction (12 out of 186) of the identified loops [15]. This suggests that additional factors or features must contribute to the formation and specificity of chromatin loops. One such candidate factor is the PRC1 component PH, since looping may be mediated via oligomerization of its SAM domain. This domain is crucial for the condensation of individual Polycomb domains [25], and for mediating long-range Polycomb domain interactions in mammals [26–28]. Furthermore, PREs in their transcriptionally active state (marked by lower levels of PcG proteins but retention of PH and GAF) can still form loops, suggesting a key function of these two factors for PRE looping [14].

An important experimental bottleneck in studying the direct contribution of GAF in chromatin looping is that it has a plethora of functions (reviewed in [29]) and is also implicated in PcG recruitment [12]. Therefore, depleting GAF levels or analyzing GAF mutants that affect its binding affinity to chromatin, as it is the case of the POZ mutant [15], affects pleiotropic activities and prevents disentangling the direct contribution of GAF to loop formation. To overcome these limitations, we combined two complementary experimental approaches. First, we assess genome-wide correlations between GAF binding levels, motif content, and chromatin looping across development by performing CUT&RUN and Micro-C profiling in *Drosophila* embryos and larval imaginal discs. We then used CRISPR/Cas9 genome engineering to either mutate GAF binding motifs or replace a PRE in the *dac* Polycomb domain with sequence variants that differentially recruit GAF and PcG proteins, enabling us to uncouple their binding without affecting global GAF function. Our results show that, although high levels of GAF and PH are hallmarks of PRE loops, GAF is not sufficient to drive looping on its own and requires additional factors. Surprisingly, PH is also dispensable for PRE looping. Moreover, exchanging PRE sequences with homologous, orthologous, or unrelated PRE sequences differentially affects PRE looping. Together, these findings indicate that PRE looping cannot be explained solely by the presence or abundance of a single factor. Instead, they suggest that specific sequence features and combinatorial recruitment of multiple factors contribute to the establishment and robustness of PRE interactions.

By analogy to PcG recruitment to PREs, which involves multiple factors, we propose that PRE looping likely depends on the combined action and relative dosage of several looping-associated factors at loop anchors. Such a combinatorial mechanism could contribute both to loop formation and to interaction specificity between PREs. While further work will be required to define the precise molecular determinants, our results are consistent with the existence of locus-specific rules that influence PRE looping behavior.

## Materials and methods

### Fly work and generation of mutant flies by CRISPR/Cas9 genome engineering

All flies were raised on standard corn meal yeast extract medium at 25°C. CRISPR/Cas9 mutant fly lines ΔPRE2 and (GA)n_mut6 are described in [12]. Sequences of guide RNAs (gRNAs) used to create fly lines Hsp26, Hsp26 + 3xPHO, (GA)n_mut2 and (GA)n_mut4, and PRE replacement lines (*Vir, Fab-7*, and *en*) are described in Supplementary Table S2. Sense and antisense oligonucleotides were annealed and phosphorylated by the T4 polynucleotide kinase (NEB #M0201S) before being inserted inside a pCFD3 plasmid (Addgene #49 410) previously digested by BbsI (NEB #R0539S). pHD-dsRED donor plasmid (Addgene) containing a removable (floxed) 3XP3-dsRED construct flanked by loxP sites and DNA fragments and having homology arms to the *dac* transcription start site (TSS) region serving as template for homology-directed repair, is described in [12]. Primer sequences to insert homology arms are described in Supplementary Table S2.

The *Hsp26* sequence and the *dac* TSS PRE WT sequence were amplified from genomic DNA and inserted into the pHD-dsRED donor plasmid cut by SpeI and BglII using GIBSON cloning Supplementary Table S2). These plasmids were used as DNA template for Multi Site-Directed Mutagenesis to generate sequences Hsp26 + 3xPHO (STRATAGENE, #200 514) or (GA)n_mut2 and (GA)n_mut4 plasmids (Agilent Quick Change Lightning Kit #210515-5) according to manufacturer instructions. PRE sequences from the *engrailed* gene (*en*), *Fab-7*, or virilis PRE (*vir*) were amplified from genomic DNA of *Drosophila melanogaster* or *Drosophila virilis* and inserted into the pHD-dsRED donor plasmid cut by SpeI and BglII using GIBSON cloning Supplementary Table S2).

To generate mutant fly lines, gRNA-containing pCFD3 and pHD-dsRED donor plasmids were injected into flies expressing Cas9 in the germline [vas-Cas9(X) RFP-; Bloomington stock #55 821]. Injections and dsRED screening was performed by BestGene (https://www.thebestgene.com/). To remove the dsRED reporter construct, mutant flies were crossed with a fly line expressing CRE recombinase (Bloomington stock #34 516). Genotypes of mutant fly lines were confirmed by polymerase chain reaction (PCR) genotyping and sequencing analysis of the mutated region. The thermosensitive RNA interference (RNAi) system to knock down PH in eye imaginal discs is described in detail in [30]. Briefly, ph-RNAi is under control of UAS sequences. Cells expressing ey-FLP induce FLP-out of a transcriptional stop (located between two flippase recognition target [FRT] sites) in eye imaginal discs, leading to expression of act-Gal4. tub-Gal80ts encodes a ubiquitously expressed, temperature-sensitive Gal4 repressor. At 18°C Gal80ts represses Gal4 and the RNAi is not expressed. At restrictive temperature (29°C), Gal80ts is inactivated. Gal4 activates UAS sequences, expressing ph-RNAi. Control flies were grown at 18°C and dissected 10 days after egg laying (AEL). To knock down PH, flies were kept at 29°C throughout development and dissected 5 days AEL.

### CUT&RUN experiments

CUT&RUN experiments were performed as described by Kami Ahmad in protocilas.io (https://dx.doi.org/10.17504/protocols.io.umfeu3n) with minor modifications. A total of 16–20 h *Drosophila* embryos were dechorionated using bleach for 2 minutes and then homogenized in 1 ml of nuclear extraction buffer (20 mM HEPES, 10 mM KCM, 0.5 m M spermidine, 0.1% Triton X, 20% glycerol, and protease inhibitor cocktail) using a Glass douncer homogenizer. The embryo homogenates were centrifuge for 3 min at 700 × *g* and washed with nuclear extraction buffer before the addition of Concanavalin A-coated beads. A total of 20 third instar imaginal eye or wing discs were dissected in Schneider medium, centrifuged for 3 min at 700 × *g* and washed twice with wash + buffer before addition of Concanavalin A-coated beads. MNase digestion (pAG-MNase Enzyme from Cell Signaling) was performed for 30 min on ice. After ProteinaseK digestion, DNA was recovered using SPRIselect beads and eluted in 50 μl TE. DNA libraries for sequencing were prepared using the NEBNext^®^ Ultra™ II DNA Library Prep Kit for Illumina. Sequencing (paired-end sequencing 150 bp, ∼2 Gb/sample) was performed by Novogene (https://en.novogene.com/).

### CUT&RUN analysis

CUT&RUN data analysis was essentially performed as previously described in [31]. Briefly, the sequencing read quality was assessed using FastQC and reads were aligned to the *D. melanogaster* dm6 reference genome using Bowtie 2 (v 2.4.2) [32] with the following parameters: –local —very-sensitive-local –no-unal –no-mixed –no-discsordant –phred33 -I 10 -X 700. The SAM alignment files were compressed into BAM files using the SAMtools (v1.16.1) software [33]. Sambamba markdup (v 1.0.0) [34] was used to removed duplicate reads with parameters: -r –hash-table-size 500 000 –overflow-list-size 500000. The peak calling was performed with each replicate as a separate input file and IgG as the control library using MACS3 (v 3.0.0b1) [35] with the following parameters: -g dm -f BAMPE -q 0.001. Highly confident GAF peaks were defined as peaks with a MACS3 score of >100 and a fold-change of >2. Mapping and peak annotation statistics are shown in Supplementary Table S1. The overlap between the GAF peaks and loop anchors was determined using the GenomicRanges package in R (v 1.58.0) [36]. Reads per kilobase per million mapped reads-normalized bigWig binary files were generated using the bamCoverage function from deepTools2 (v 3.5.5) [37]. Genome browser plots were generated using the pyGenomeTracks package (v 3.8) [38] and heatmaps using the plotHeatmap function from deepTools2.

### Motif analysis

Motif scanning analysis on GAF peaks was performed using FIMO software (v 5.5.5) [39] with the MA0205.2. and GAGAGAGAGAGAGAGAGAGAGAGAGAGAG (GA29) MEME-1 GAF motif matrices. Default parameters were used and only matched sequences with a q-value ≤0.05 were considered.

### Comparative sequence analysis and motif discovery for *dac* PRE2 orthologs

To identify homologous PRE sequences, the *dac_TSS, engrailed (en)*, and *Fab-7* PRE sequences from *D. melanogaster* were used as queries in BLAST searches against the genomes of all *Drosophila* species available at FlyBase (release FB2025_05, 11 December 2025) using default parameters. Among significant alignments (E-value < 0.05), sequences were considered homologous if they produced either a single hit covering, or multiple hits spanning, at least 75% of the query sequence. The corresponding FASTA sequences were used for motif discovery to identify sequence features specific to the *dac_TSS* PRE relative to the *en* and *Fab-7* PREs. Motif analysis was performed using MEME software (v 5.5.9) in Discriminative Mode [40], with *dac_TSS* homologous sequences as the primary set and *en* and *Fab-7* homologous sequences as the control set. The following parameters were used: Zero or One Occurrence Per Sequence (ZOOPS), a maximum of 5 motifs, and motif widths ranging from 6 to 20 bp. The top five enriched motifs identified by MEME were subsequently analyzed using Tomtom (v 5.5.9) [41] to compare them against known transcription factor (TF) binding motifs in the JASPAR database (JASPAR2026_CORE_insects_non-redundant). Matches with E-values < 0.05 were considered significant. Among the discovered motifs, only the repetitive CA motif (CACACACACACACACACA; MEME motif 5) showed a significant match, corresponding to the Combgap binding motif (MA2107.1).

### *k*-Means clustering of histone marks, PH profiles, and ATAC-seq data around GAF peaks

GAF peaks identified in embryo, larval eye or wing imaginal discs, or the merged dataset (for developmental comparisons; see Supplementary Fig. S3) were used to analyze signal tracks around the peak center. Signal intensities were normalized and stratified into a defined number of clusters using the *kmeans* function in R (version 4.4.3; www.R-project.org). Heatmaps and average signal profiles were generated using the seqplots R package (v1.23.3) [42].

### Source of CUT&RUN, ChIP-seq, and ATAC-seq data

CUT&RUN data for H3K27me3, H3K27ac marks and ChIP-seq for PH data in control and PH-depleted eye imaginal discs are from [30] (GEO accession number GSE222193). The ChIP-seq data for PH in embryos was obtained from [43]. The ATAC-seq data from 14- to 17-h embryos were obtained from [44], and the data for the imaginal wing discs were obtained from the 0 HS control condition in [45]. The bigwig files for the two replicates were downloaded from the Gene Expression Omnibus database (accession numbers GSE120150 and GSE102839, respectively). The average of the replicates was then calculated using the bigwigAverage function from deepTools2.

### Hi-C experiments

Hi-C experiments were performed using the EpiTect Hi-C Kit (Quiagene #59 971). All Hi-C experiment were performed in two or three independent experiments using 50 third instar imaginal discs. Briefly, discs were homogenized and fixed in activated Buffer T and 2% Formaldehyde using Tissue Masher tubes [Biomasher II (EOG-sterilized) 320 103 Funakoshi]. Tissue was digested by adding 25 μl Collagenase I and II (40 mg/ml) for 1 h at 37°C. Samples were centrifuged and supernatant was carefully aspirated, leaving ∼250 μl of solution in the tube. Then 250 μl QIAseq Beads equilibrated to room temperature were added to bind nuclei to the beads and all subsequent reactions were performed on the beads according to the manufactures protocol. Libraries were sequenced at BGI (https://www.bgi.com/) PE 150. Sequencing statistics are summarized in Supplementary Table S1.

### Micro-C experiments

Micro-C experiments were performed using the Dovetail Micro-C Kit (#21 006) according to manufacturer instructions with the following modifications. About 200 *Drosophila* embryos or 50 imaginal discs were collected or dissected into BioMasher II tube (PeloBiotech, PN: 320 103) and snap frozen in liquid N2 and stored for at least 30 min at −80°C.

After thawing embryos or imaginal discs, they were resuspended in 150 μl phosphate buffered saline (PBS) + 0.3 M Disuccinimidyl glutarate (DSG) (freshly prepared) and homogenized with the pestel provided with the BioMasher II tubes. After material is completely homogenized, additional 850 μl PBS + 0.3 M DSG is added and tubes are rotated at room temperature for a total time of 10 min. Then 27 μl of 37% formaldehyde are added for additional 10 min. After fixation, nuclear pellet is washed twice with 1 ml 1× Wash Buffer. After the second wash, the pellet is resuspended again in 1 ml 1× Wash Buffer and using a 1 ml syringe, gently pushed through a 50 μm filter into a new 1.5 ml tube. After centrifugation, the nuclear pellet is resuspendend in 50 μl 1× Nuclease Digest Buffer. Embryonic nuclei were digested for 15 min at 22°C with 0.5 μl MNAse (undiluted), whereas nuclear pellet from imaginal discs was digested with 0.5 μl MNAse (1:10 diluted). Mnase digestion was stopped by adding 5 μl 0.5 M Ethylene Glycol Tetraacetic Acid (EGTA). For embryo Micro-C about 1500 ng of the lysate was used as Input for Micro-C procedure. For discs Micro-C the whole lysate was used.

Library preparation was performed using theNEBNext^®^ Ultra™ II DNA Library Prep Kit for Illumina (12 cycles, DNA Polymerase from Dovetail Kit). All Micro-C experiments were performed in duplicates. Libraries were sequenced at BGI (https://www.bgi.com/) PE 100. Sequencing statistics are summarized in Supplementary Table S1.

### Hi-C and Micro-C analysis

**Figure 1.**
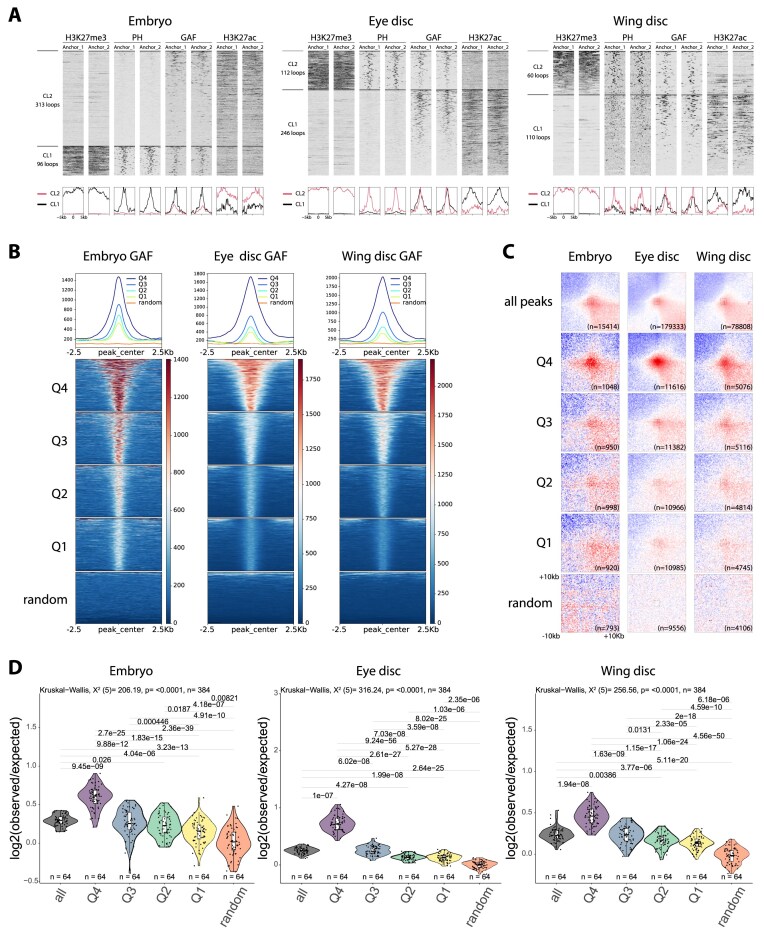
GAF binding strength correlates with chromatin looping. (**A**) Heatmaps and average signal profiles showing *k*-means clustering of H3K27me3, PH, GAF, and H3K27ac signals around the anchors (1 and 2) of the loops detected in Micro-C of embryos (left) larval eye discs (middle) and larval wing discs (right). (**B**) Heatmaps and average signal profiles of GAF CUT&RUN (two merged replicates) centered on GAF peaks ranked and grouped into quartiles based on the MACS3 peak score. Random peaks matched in number and average size were used as control. (**C**) Aggregate Micro-C map at 250 bp resolutions around pairs of GAF peaks, which are grouped as in panel (B) at distance separation between 10 and 500 kb. (**D**) Violin plots show the the contact-enrichment values in a 8 × 8 square in the center of the aggregate Micro-C maps presented in panel (C). Boxplots show median (central line), Q1 = 25th and Q3 = 75th percentiles (box limits), and Q1 + 1.5 × IQR to Q3 + 1.5 × IQR (whiskers). Statistical comparisons were performed using the Kruskal–Wallis test followed by Dunn’s *post-hoc* pairwise test with Benjamini–Hochberg correction.

**Figure 2.**
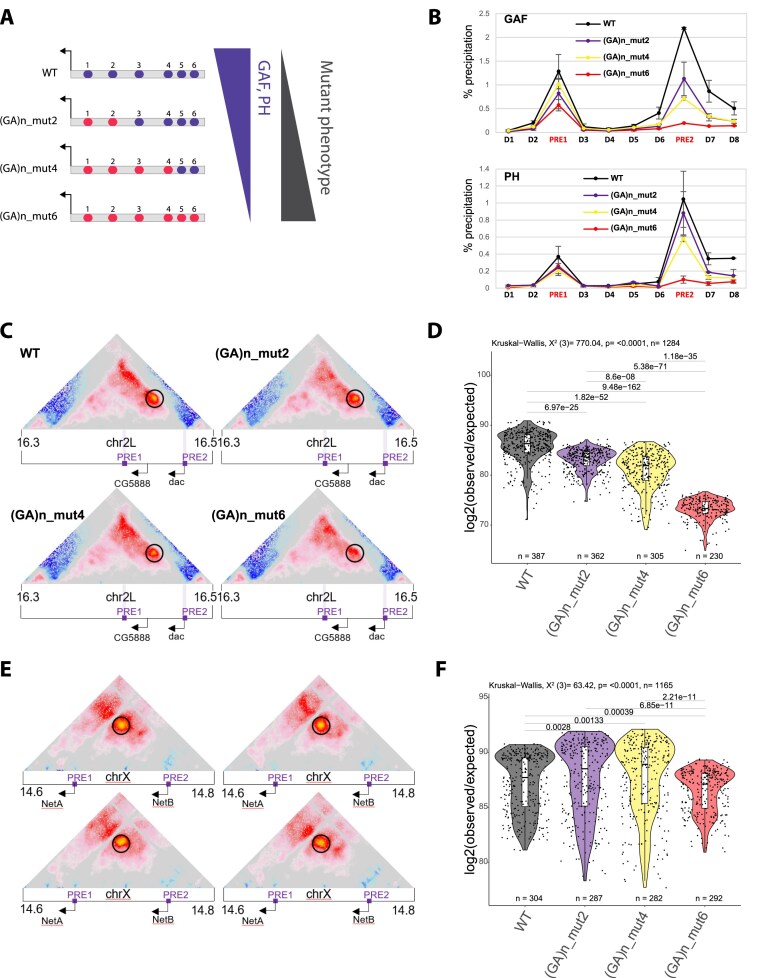
GAF binding levels are a major determinant for PRE loop formation. (**A**) Schematic representation of the PRE2 with (GA)n motifs (GAF binding sites) shown in blue. Mutated motifs are indicated in red *(left)*. Schematic representation of the consequences of mutating increasing numbers of (GA)n motifs on the binding of indicated chromatin factors (*right*). (**B**) qChIP experiments using GAF or PH antibodies in WT, (GA)n_mut2, (GA)n_mut4, and (GA)n_mut6 fly lines. D1–D8, PRE1, 2 indicate PCR amplicons used for qChIP experiments along the *dac* gene locus (Supplementary Table S2). The housekeeping gene *Rp49* was used as negative control, the *engrailed* PRE as a positive control. Data are presented as the mean values ± standard deviation (SD) (error bars) of two independent replicates. (**C, E**) Micro-C score (see the ‘Materials and methods’ section) maps of a 200 kb region at 1 kb resolution on chromosome 2L at *dac* gene locus (**C**) and on chromosome X at the *NetA/B* gene locus (**E**) in WT, (GA)n_mut2, (GA)n_mut4, and (GA)n_mut6 fly lines. Black circles indicate the positions of the PRE loop. Violet bars indicate position of PREs. Black arrows indicate gene promoters of the *dac, CG5888, NetA*, and *NetB* genes. (**D, F**) Quantification of the Micro-C *dac* (**D**) and *NetA/B* (**F**) PRE loop interaction scores in WT, (GA)n_mut2, (GA)n_mut4, and (GA)n_mut6 fly lines. Violin plots show the distribution of Micro-C scores for each of the contacts (pair of chromatin fragments) falling between the anchors of the interrogated loop. Violin plots show median (central line), Q1 = 25th and Q3 = 75th percentiles (box limits), and Q1 + 1.5 × IQR to Q3 + 1.5 × IQR (whiskers). Statistical comparisons were performed using the Kruskal–Wallis test followed by Dunn’s *post-hoc* pairwise test with Benjamini–Hochberg correction.

**Figure 3.**
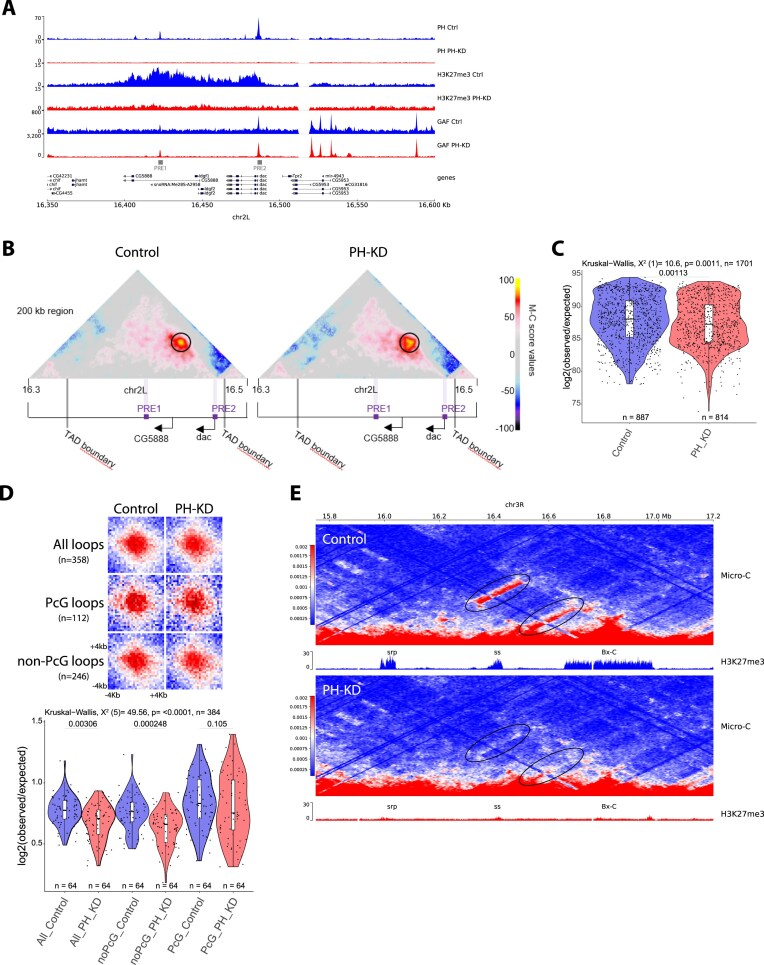
PH is not necessary for PRE looping. (**A**) ChIP-seq profiles for PH and CUT&RUN profiles H3K27me3 and GAF in Control (Ctrl) or PH-depleted (PH-KD) larval eye imaginal discs at the *dac* gene locus. (**B**) Micro-C score maps of a 200kb region around the *dac* TAD in control or PH depleted (PH KD) larval eye imaginal discs. PRE loop is indicated by a black circle. Violet bars indicate position of PREs. Gray bars indicate the positions of TAD boundaries. Black arrows indicate the promoters of the *dac* and the *CG5888* genes. (**C**) Quantification of the *dac* PRE loop Micro-C interaction scores in Control and PH depleted (PH-KD) mutant flies. Statistical comparisons were performed using the Kruskal–Wallis test followed by Dunn’s *post-hoc* pairwise test with Benjamini–Hochberg correction. (**D**) Aggregate Micro-C map at 250 bp resolutions centered at *All, PcG*, and *non-PcG* loops in Control and PH depleted (PH-KD) Micro-C (see Supplementary Table S1 and the ‘Materials and methods’ section). Violin plot shows the quantification of the signal in the central 8 × 8 square of the aggregate Micro-C maps. Statistical comparisons were performed using the Kruskal–Wallis test followed by Dunn’s *post-hoc* pairwise test with Benjamini–Hochberg correction. Boxplots in panels (**C, D**) show median (central line), Q1 = 25th and Q3 = 75th percentiles (box limits), and Q1 + 1.5 × IQR to Q3 + 1.5 × IQR (whiskers). (**E**) Micro-C contact maps in control eye discs (top) or upon loss of PH function (PH-KD, bottom) showing the loss of long range contacts between Polycomb domains.

**Figure 4.**
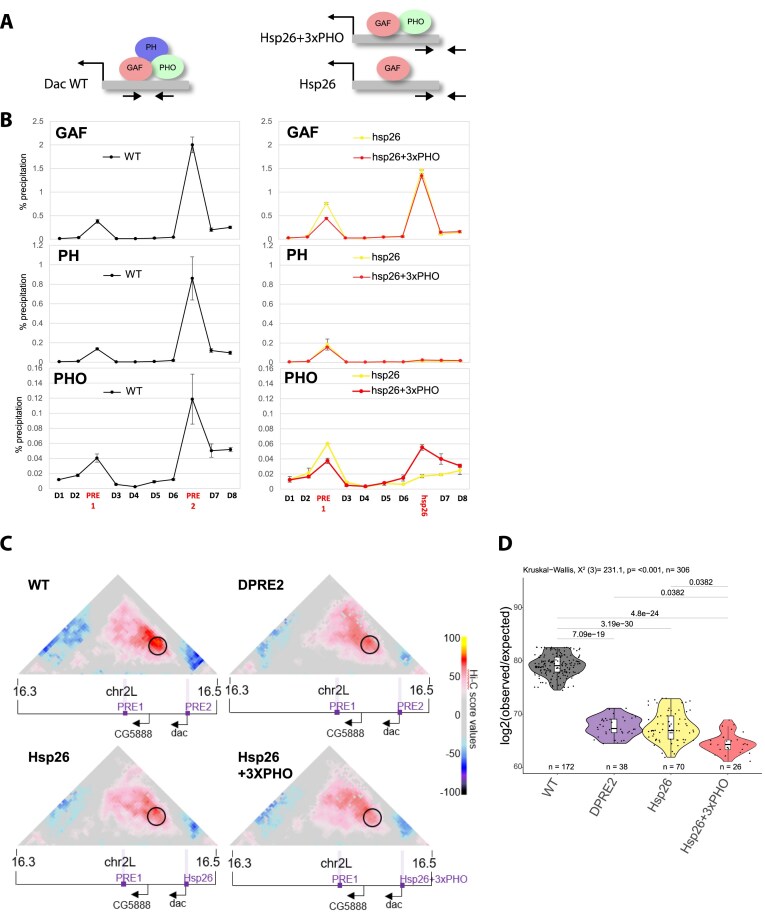
GAF is not sufficient for PRE loop formation. (**A**) Schematic representation of the Hsp26 constructs inserted at the *dac* TSS (PRE2) genomic region. Location of primer pairs for qChIP are indicated by arrows (**B**) qChIP experiments using GAF, PH, or PHO antibodies in WT, Hsp26, and Hsp26 + 3xPHO fly lines. D1–D8, PRE1, 2 indicate PCR amplicons used for qChIP experiments along the *dac* gene locus (Supplementary Table S2). Note that, to discriminate endogenous *Hsp26* sequence from the ectopically inserted sequence, we used a primer pair, where one primer is specific to the *Hsp26* sequence whereas the other one is specific to the *dac* promoter region. The housekeeping gene *Rp49* was used as negative control, the *engrailed* PRE as a positive control. Data are presented as the mean values ± SD (error bars) of two independent replicates. (**C**) Hi-C score (see the ‘Materials and methods’ section) maps of a 200 kb region at 3 kb resolution on chromosome 2L at *dac* gene locus in imaginal discs in WT, PRE2 deleted (ΔPRE2), Hsp26, and Hsp26 + 3xPHO fly lines. Black circle indicates the position of the *dac* PRE loop. Violet bars indicate position of PREs. Black arrows indicate gene promoters of the *dac* and the *CG5888* genes. (**D**) Quantification of the *dac* PRE loop interaction scores. Hi-C interaction score in WT, PRE2 deleted (ΔPRE2), Hsp26, and Hsp26 + 3xPHO fly lines. Boxplots show median (central line), Q1 = 25th and Q3 = 75th percentiles (box limits), and Q1 + 1.5 × IQR to Q3 + 1.5 × IQR (whiskers). Statistical comparisons were performed using the Kruskal–Wallis test followed by Dunn’s *post-hoc* pairwise test with Benjamini–Hochberg correction.

**Figure 5.**
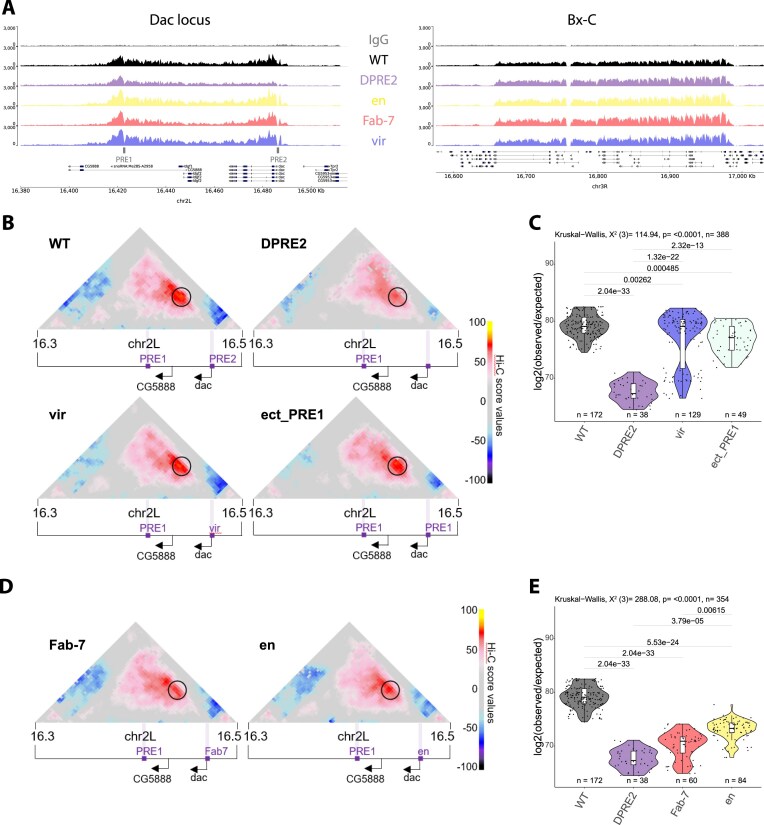
An orthologous PRE sequence can rescue loss of PRE looping. (**A**) CUT&RUN profiles of H3K27me3 around the *dac* gene (left) or the Bx-C locus (right) in WT, PRE2 deleted (ΔPRE2), or mutant PRE lines, where the *dac* PRE2 has been replaced by the indicated PREs (*en, Fab-7* or *vir*). (**B, D**) Hi-C score (see the ‘Materials and methods’ section) maps of a 200 kb region at 3kb resolution on chromosome 2L at *dac* gene locus in imaginal discs in WT, PRE2 deleted (ΔPRE2), or mutant PRE lines, where the *dac* PRE2 has been replaced by the indicated PREs (*vir*, ect_PRE1 and *Fab-7, en*). Black circle indicates the position of the *dac* PRE loop. Violet bars indicate position of PREs. Black arrows indicate gene promoters of the *dac* and the CG5888 genes. (**C, E**) Quantification of the *dac* PRE loop interaction scores. Hi-C interaction score in WT, ΔPRE2, or the mutant PRE lines *vir*, ect_PRE1, *Fab-7*, and en. Statistical comparisons were performed using the Kruskal–Wallis test followed by Dunn’s *post-hoc* pairwise test with Benjamini–Hochberg correction on all the sample considered together as in Supplementary Fig. S9F. Boxplots show median (central line), Q1 = 25th and Q3 = 75th percentiles (box limits), and Q1 + 1.5 × IQR to Q3 + 1.5 × IQR (whiskers).

Raw data from Hi-C sequencing were processed using the ‘scHiC2’ pipeline and from Micro-C with a modified version to filter-out contacts at distance separation lower than 200 bp. The samples for Embryo_WT, WD_WT, (GA)n_mut, and larvae_DWT were aligned to the *D. melanogaster* reference genome dm6 in https://s3.amazonaws.com/igenomes.illumina.com/ and samples for the *dac* PRE2 mutants (ΔPRE2, *vir*, ect_PRE1, *Fab-7*, and *en*) were aligned to a modified genome. Briefly, the 644 bp region chr2L:16 485 929–16 486 572 including the PRE2 was removed from the dm6 reference genome and replaced with strings corresponding to the specific mutation (Supplementary Table S2). For all mutations except for ect_PRE1, we added a sequence of N (indetermined) nucleotides to replace entirely the removed sequence of 644. For ect_PRE1 mutant, we cut the end of chr2L by 459 bp to maintain its total length of 23 513 712 bp, as for the wild type (WT). To improve mappability, the *.fastqs* of Micro-C reads were trimmed to 100 nt (if necessary) using trim_galore (https://github.com/FelixKrueger/TrimGalore). Sequencing statistics are summarized in Supplementary Table S1. Valid-pairs were stored in a database using the ‘misha’ R package (https://github.com/msauria/misha-package). Extracting the valid interactions from the misha database, the ‘shaman’ R package (https://bitbucket.org/tanaylab/shaman) was used for computing the Micro-C and Hi-C expected models and scores with parameters k = 250 and k_exp = 500 (Figs 2C and E, 3B, 4C, and 5B and D, and Supplementary Fig. S5B). Specifically, Micro-C and Hi-C scores quantify the contact enrichment (positive values) or depletion (negative values) of each fragment-pair with respect to a statistical model used to evaluate the expected number of counts. In the experimental procedures, these fragments are generated by digestion (by restriction fragments in Hi-C or by MNase in Micro-C) and then are re-ligated to create the pair. To generate the expected model, we randomized the observed Hi-C contacts using a Markov chain Monte Carlo-like approach per chromosome [46]. Shuffling was conducted such that the marginal coverage and decay of the number of observed contacts with the genomic distance were preserved but any features of genome organization (for example, TADs or loops) were not. These expected maps were generated for each biological replicate separately and contained twice the number of observed *cis* contacts. Next, the score for each contact in the observed contact matrix was calculated using the *k*-nearest neighbors (kNN) strategy [46]. In brief, the distributions of 2D Euclidean distances between the observed contact and its nearest k_exp neighbors in the pooled observed and pooled expected (per cell type) data were compared, using Kolmogorov–Smirnov D statistics to visualize positive (higher density in observed data) and negative (lower density in observed data) enrichments. These D scores were then used for visualization (using a scale from −100 to + 100) and are referred to as Hi-C scores in the text. Accordingly, the color scale of the Hi-C scores comprises both positive and negative values. The maps of Micro-C and Hi-C scores show a value per bin. For a given bin *b*, the score value is the average of the scores of all the fragment-pairs falling in a *b*. For each condition, the Hi-C and Micro-C interaction quantifications at PRE loops (Figs 2D and F, 3C, 4D, and 5C and E, and Supplementary Figs S5C and S9F) were performed by considering the Hi-C scores of any pair of contact found between two regions of 6 kb, chr2L:16 419 514–16 425 515 and chr2L:16 482 929–16 488 930), including the *dac* PRE1 and PRE2 or two regions of 6 kb, chrX:14 650 251–14 659 252 and chrX:14 748 251–14 754 251 including the NetA/NetB PREs (Fig. 2E), respectively. To measure the total enrichment of contacts, between pairs of GAF peaks whose centers are separated by at least 10 kb and not >500 kb (Fig 1C, and Supplementary Figs S3B and 4A–C) and identified loops an enrichment analysis was applied. Briefly, the log_2_(Observed/Expected) score in squared windows of size *w* centered at the considered features (*w *= 20 kb for GAF peaks and *w *= 8 kb for Micro-C loops) at 250 bp resolutions was computed using the *shaman_generate_feature_grid_2d* function of the Shaman package. The box plots in the distributions of Hi-C and PE-scan enrichment scores (Figs 1C and D, and 3D, and Supplementary Figs S3B and 4A–C) show the median (central line), the 75th and 25th percentiles (box limits) and 1.5 × the interquartile range (IQR; whiskers). Statistical comparisons between distributions of Hi-C and contact enrichment scores per conditions were performed using the Kruskal–Wallis test followed by Dunn’s *post-hoc* pairwise test with Benjamini–Hochberg correction. *Adjusted P-values* <.05 are reported on top of the compared distributions.

### Loop-calling analysis

Loop-calling analysis was performed on .mcool files which were obtained and normalized via the Iterative Correction and Eigenvector decomposition algorithm (ICE) with default parameters (command “*cooler zoomify -r 400, 800, 1000, 2000, 4000, 8000, 10 000, 20 000, 40 000 file.cool -o file.mcool –balance*”). To call loops from Micro-C maps, the following strategy as been applied. (i) Firstly, manually-curated a sets of *Gold Standard* loops per condition have been obtained from the set of loops published in Ref. [16]. This step has been carried out by plotting the Micro-C maps at different resolution. (ii) The *mustache* loop-caller [47] has been applied to detect loops on matrices of different resolutions with specific ranges of minimum and maximum distances between the anchors. Loop-detection has been done at resolutions 400, 800, 1000, 2000 bp between 0 and 400 kb; 800, 1000, 2000, and 4000 bp between 0 and 800 kb; 4000, 8000, 10 000, and 20 000 bp between 0 and 3.2 Mb, and 20 000 and 40 000 bp between 0 and 33 Mb. We applied *mustache* with the following *mustache* parameters *sparsityThreshold* = 1.00, *iteration* = 5, and *sigmaZero* varying between 0.6 and 3.6 every 0.1. (iii) Loops in the pericentromeric regions (chr2L:22 113 700–23 513 712, chr2R:1–5 756 000, chr3L:22 933 988–28 110 227, chr3R:1–4 027 467, chrX:21 572 709–22 396 687, and chrX:22 929 901–23 542 271) were filtered out because they were detected in regions with poor mappability. (iv) Next, for each of the resolutions, sets of loops with false discovery rates (FDR) smaller than a threshold value which was set from 0.0001 to 0.1 increasing by steps of 0.0001. Loops closer than one bin were merged (command: *PyGLtools/merge.py -d resolution*). (v) To evaluate the optimal FDR per resolution, we computed how many of the Gold Standard [defined at point (i)] were detected and defined the ratio between the number of Gold Standard loops detected and the total number of loops detected (r_GS_). We finally set for each resolution the value of FDR that allows us to maximize the r_GS_. This results in a set of loops per resolution. (vi) Finally, the union of all the loops that pass the FDR threshold per resolution were inspected visually. To ease this visual inspection, we plotted the entire region spanned by the loops (±25 bins) and marked the putative loop on the matrix using a dashed square. (vii) After manual inspection, we merged the accepted loops for different resolutions where the anchors have different sizes. Each of these loops were recentered on the location within the looping area with the highest Micro-C score: the final set of loops is obtained by fixing the location of the maximum ±2 kb. This recentering is motivated by the fact that the Shaman score remove the local genomic-distance bias and help to locate the summit of the loops. (viii) In conclusion, we obtained: 358 loops from Micro-C in ED_Control, 409 loops from Micro-C in Embryo_WT, and 170 loops from Micro-C in WD_WT (Supplementary Table S1). Signals for H3K27me3, PH, GAF, and H3K27ac around paired loop-anchors were used to cluster loops (Fig. 1A). Signal intensities were normalized and stratified into a defined number of clusters (k = 2) using the *kmeans* function in R (version 4.4.3; www.R-project.org). Heatmaps and average signal profiles were generated using the seqplots R package (v1.23.3) [42].

### Quantitative chromatin immunoprecipitation (qChIP) experiments

qChIP experiments were performed as described in [9] with minor modifications. Chromatin was sonicated using a Bioruptor Pico (Diagenode) for 7 min (30 s in, 30 s off). All antibodies were diluted 1/100 for the immunoprecipitation. After decrosslinking, DNA was purified using MicroChIP DiaPure columns from Diagenode. Enrichment of DNA fragment was analyzed by real-time PCR Light-cycler 480 (Roche). Primers used are indicated in Supplementary Table S2. PHO antibody is a generous gift of J. Kassis [48]. Psq antibody is a generous gift from CA Berg [49]. GAF, PH, E(Z) antibodies are described in [12]. H3K27me3 antibody is from ActiveMotif (#39 155).

## Results

### GAF binding levels correlate with chromatin looping genome-wide

To investigate the genome-wide relationship between GAF occupancy and chromatin looping, we performed CUT&RUN targeting GAF, the PRC1 subunit Polyhomeotic (PH), active and repressive histone marks (H3K27Ac and H3K27me3, respectively) alongside high-resolution Micro-C, in *Drosophila* embryos (16–20 h) and in two distinct third instar larval tissues: imaginal eye and wing discs (Supplementary Table S1). We identified 1855, 6441, and 4272 high-confidence GAF peaks in embryos, larval imaginal eye and wing discs, respectively. Micro-C analysis revealed 409 high-confidence loops in embryos, 358 in eye imaginal discs and 170 in wing imaginal discs (Supplementary Fig. S1), with median loop sizes of 59, 45, and 71 kb, respectively (Supplementary Fig. S2A). Among the 409 chromatin loops identified in embryos, 81 are shared with both larval tissues, 70 are shared exclusively with eye discs, and 9 are shared exclusively with wing discs. In total, 160 loops (39%) are retained in at least one larval tissue (Supplementary Fig. S1). While the remaining 61% were identified only in embryos and are therefore classified as embryo-specific, these loops are not entirely absent in other tissues but instead display reduced interaction frequencies, indicating quantitative modulation rather than complete loss. Overall, these results support a model in which a substantial fraction of chromatin loops is maintained across development, as exemplified by the previously characterized PRE loop at the *dac* locus [13], while another fraction is more dynamically regulated, primarily through changes in interaction strength rather than complete disappearance. Although only a small subset of all GAF peaks engages in looping interactions (Supplementary Fig. S2B), as also reported in [21], GAF binding is a general hallmark of chromatin loops (Fig. 1A), consistent with previous reports [4, 12]. In embryos, 38% of loops overlap with GAF peaks, with roughly half showing GAF binding at both anchors and half at a single anchor. At the larval stage, this fraction increases to 70% in wing imaginal discs and 85% in eye imaginal discs, with most of these loops bound by GAF at both anchors (Supplementary Fig. S2C and Supplementary Table S1). These findings suggest that GAF involvement in looping becomes more prominent during development. The median sizes of GAF-overlapping loops are comparable between stages, 43 kb in embryos and 39 or 46 kb in eye or wing imaginal discs (Supplementary Fig. S2A). Importantly, chromatin loop-anchors located within repressive H3K27me3 domains exhibit a particularly strong association with GAF binding: 71%, 92%, and 83% of H3K27me3-enriched loops in the embryo, eye and wing imaginal discs, respectively, overlap with GAF peaks in at least one loop anchor. These anchors are also bound by PH (Fig. 1A), highlighting a predominant role for GAF in mediating chromatin loops between PREs.

Notably, GAF sites associated with loop anchors exhibited significantly higher occupancy than at nonlooping sites (Supplementary Fig. S2D), suggesting a quantitative relationship between GAF occupancy and loop formation. To investigate this further, we ranked all GAF peaks based on their peak scores, used as a proxy for binding strength, and grouped them into quartiles where Q4 (Q1) had the highest (lowest) GAF binding strength (Fig. 1B). This analysis revealed a skewed distribution of GAF binding, with high-occupancy GAF peaks more likely to overlap with loop anchors (Supplementary Fig. S2E). Consistent with previous reports showing that GAF binds preferentially to clustered (GA)n repeats via its POZ domain [17, 50], we also found that stronger GAF peaks were associated with both larger peak sizes (Supplementary Fig. S2F) and higher numbers of underlying GAF motifs (Supplementary Fig. S2G and H). To directly test whether GAF binding levels influence loop formation, we focused on pairs of GAF peaks within 500 kb of genomic separation, since the majority of GAF-associated loops fall within this range (Supplementary Fig. S2A). Importantly, this analysis confirmed a strong positive correlation between GAF occupancy and the likelihood of looping: the higher the GAF peak score, the greater the looping score, hence the greater the tendency for the site to engage in chromatin loops (Fig. 1C and D).

Together, these results demonstrate that GAF binding strength is a robust predictor of chromatin looping independently of the examined tissue and support a model in which GAF occupancy plays a critical role in establishing chromatin loops during *Drosophila* development.

Interestingly, a similar positive relationship between protein binding levels and chromatin contact frequency has previously been reported for PRC1 components, including PH [51]. To explore the chromatin landscape of GAF binding sites involved in looping, we clustered CUT&RUN profiles of the H3K27ac and H3K27me3 histone marks, together with PH and chromatin accessibility (ATAC-seq) data in all GAF peaks identified in both embryos and larval imaginal eye and wing discs (Supplementary Fig. S3). Strikingly, the cluster (CL) with the strongest chromatin looping between GAF peaks also showed high levels of PH binding (Supplementary Fig. S3A and B, CL4). Moreover, genomic regions that gain GAF binding at the larval stage compared to embryos display a significant increase in chromatin looping (Supplementary Fig. S3A and B, CL3). This increase is followed by elevated PH occupancy and enhanced chromatin accessibility, suggesting a coordinated change in chromatin state. Of note, GAF and PH co-localization and strong binding at H3K27me3 domains was associated to the strongest loops in embryos, but in larval tissues an even stronger looping was observed in a cluster of sites associated with H3K27Ac (Supplementary Figs S3 and S4), suggesting that active chromatin regions might increase looping efficiency during late stages of development. In summary, the strong correlation between GAF and PH binding levels, as well as chromatin contact frequencies at all developmental stages and examined tissues, suggests that both proteins may play a functional key role in the formation of chromatin looping.

### GAF binding levels dictate PRE loop formation and PH recruitment at the *dac* locus

Since chromatin loop anchors within H3K27me3 domains show strong enrichment for both GAF and PH binding (Fig. 1A), and GAF sites overlapping with PH in Polycomb domains establish robust chromatin loops genome-wide (Supplementary Fig. S4), we next focused our analysis on dissecting the respective contributions of GAF and PH to PRE looping. Previous work demonstrated that a PRE loop at the *dac* gene locus depends on both an intact PRE sequence and GAF binding [12]. Here, we found that deletion of the PRE sequence not only leads to the loss of GAF binding [12], but also to a significant reduction in PRE looping that correlates with the appearance of extra sex comb bristles at the second tarsal segment of male flies, a very specific gain-of-function phenotype resulting from *dac* overexpression in this tarsal segment [12, 13] (Supplementary Figs S5 and S6). To further dissect how GAF binding levels influence the formation of PRE loops, we leveraged the correlation between GAF occupancy and the number of underlying (GA)n repeat motifs (Supplementary Fig. S2). Therefore, we generated a series of mutant fly lines carrying targeted mutations in increasing numbers of (GA)n motifs within the *dac* PRE2 sequence (Fig. 2A).

qCHIP experiments confirmed that mutation of all six (GA)n motifs abolished both GAF and PH binding (Fig. 2B). Mutation of four (GA)n motifs resulted in a partial loss of GAF and PH binding, while mutation of only two (GA)n motifs led to an even lower reduction of GAF and PH levels compared to the wild type control (WT) (Fig. 2B). Therefore, this engineered allelic fly line series provides a quantitative gradient of GAF and PH binding levels to the PRE2 sequence. In contrast, two other PRE-bound TFs that either interact with GAF or bind to (GA)n motifs, Pleiohomeotic (PHO), and Pipsqueak (PSQ), can efficiently bind to the PRE2 sequence upon mutation of 2 or 4 (GA)n motifs, whereas their binding is only reduced when all (GA)n motifs are deleted (Supplementary Fig. S6A).

Next, we asked whether the strength of GAF and/or PH binding is a key determinant of PRE loop formation. To test this, we performed high resolution Micro-C experiments in the different (GA)n mutant lines and quantified the *dac* PRE loop (Fig. 2C and D). As previously observed, mutations of all (GA)n motifs led to a significant disruption of the PRE loop. Importantly, even mutation of just 2 (GA)n sites was sufficient to significantly reduce PRE looping, with the loss of PRE looping progressively increasing as more motifs were mutated and GAF/PH binding levels decreased (Fig. 2C and D). In contrast, looping frequencies between two PREs at the *NetA/B* gene locus remained unaffected by (GA)n motifs mutations at the *dac* locus, and chromatin contacts of all chromatin loops identified in embryos in not affected (Supplementary Fig. S6B), indicating that the effect is specific to the mutated PRE loop (Fig. 2E and F). This observation supports a local, locus-specific effect of (GA)n motif mutations on the targeted PRE rather than a global perturbation of chromatin looping. Strikingly, the gradient of GAF and PH binding levels upon (GA)n motifs mutations closely mirrors the penetrance of a gain-of-function phenotype of the *dac* gene (Supplementary Fig. S6C), which has been previously shown to be induced by the specific overexpression of *dac* in the TS2 segment in pupal leg discs [13]. Of note, (GA)n motifs mutations at PRE2 also slightly reduced GAF binding levels at the neighboring PRE1, which might reflect a stabilizing effect of GAF binding upon PRE looping. In summary, these results indicate that high GAF binding levels are critical for robust PRE loop formation and for buffering the expression of looping-dependent mutant phenotypes at the *dac* locus, with PH binding potentially taking a part in this process.

### PH binding is dispensable for PRE looping

To directly assess whether PH is necessary for PRE loop formation, we made use of a temperature-sensitive system, previously shown to efficiently knock down PH protein levels in imaginal eye discs [30]. Global loss of PH binding to chromatin also results in the strong reduction of H3K27me3 levels [30], whereas GAF binding at PREs remains unaffected (Fig. 3A), allowing to uncouple the respective contributions of these two chromatin factors to PRE looping. We therefore performed Micro-C experiments upon loss of PH function and quantified PRE looping at the *dac* gene locus (Fig. 3B). Contrary to the loss of GAF binding, PRE looping frequency between the two PREs at the *dac* gene locus was only slightly affected upon loss of PH binding (Fig. 3C).

To extend this analysis on a genome-wide scale, we generated aggregate plots of Micro-C signals centered on PRE loops (loops bound by PH) and compared them to loops formed in the absence of PH (non-PcG loops). Quantification of aggregated loop signals revealed a slight, though not significant, decrease in PRE looping upon loss of PH. However, a significant reduction was also observed at non-PcG loops (Fig. 3D), suggesting that the decrease in PRE looping is not a direct consequence of PH loss, but may instead reflect a global change in nuclear or chromosomal architecture, or a small systematic difference in the two Micro-C samples. Interestingly, although loss of PH does not specifically affect PRE looping within TADs, long range interactions between Polycomb domains are significantly reduced (Supplementary Fig. S7A), as exemplified by the loss long range interactions between the *HOX* gene cluster Bx-C and the *ss* and *srp* Polycomb domains (Fig. 3E), as well as between Bx-C and Antp-C (Supplementary Fig. S7B). In contrast, long range interactions between active or null domains are not affected (Supplementary Fig. S7A).

Altogether, these results indicate that, although PH is necessary for the compartmentalization of Polycomb domains and PH binding levels positively correlate with PRE loop strength, PH is not essential for efficient PRE looping. PH occupancy at PREs likely reflects its recruitment downstream of GAF binding and the establishment of Polycomb repression domains, rather than a direct architectural role in loop formation. In contrast, GAF, alone or in combination with other chromatin factors bound at PREs, emerges as a key driver of PRE looping.

### GAF is required for PRE looping but remains insufficient, even in conjunction with PHO binding

To investigate whether GAF binding alone is sufficient to mediate PRE looping, or whether additional PRE-binding proteins are required, we replaced the PRE2 sequence at the *dac* gene locus with a 370 bp fragment from the *Hsp26* promoter containing well-characterized (GA)n motifs [52]. Notably, this sequence is known to recruit high levels of GAF without acting as a PRE at its endogenous locus (Supplementary Fig. S8A). Importantly, we did not include the *Hsp26* TATA box to prevent artificial gene transcription (Fig. 4A, Hsp26 mutant). qChIP analysis confirmed that GAF was efficiently recruited to the *Hsp26* sequence inserted at the *dac* TSS (Fig. 4B). However, neither PH nor PHO was detected at this site (Fig. 4B), indicating that GAF binding alone is not sufficient to recruit PcG proteins. The absence of PRE activity/PH recruitment of the *Hsp26* sequence was also reflected by a marked reduction in H3K27me3 levels across the *dac* domain, similar to what is observed in the PRE2 deletion line (Supplementary Fig. S8B). Consistent with the loss of Polycomb-mediated repression of *dac*, the Hsp26 replacement line displayed the same gain-of-function phenotype (Supplementary Fig. S8C) previously observed upon deletion of the PRE2 sequence [12].

To assess whether GAF binding alone, without PH, is sufficient to mediate PRE looping, we performed Hi-C experiments in third instar imaginal discs from flies carrying the Hsp26 replacement construct and compared PRE interaction frequency to the WT and the PRE2 deletion lines (Fig. 4C). Quantification of interactions between the PRE1 and PRE2 regions showed that the Hsp26 line exhibits significantly reduced interaction frequencies compared to WT flies, with a similar interaction frequency than the PRE2 deletion line (Fig. 4D). These results indicate that GAF binding alone is not sufficient to mediate PRE looping interactions within Polycomb domains, suggesting that additional PRE-associated looping factors are needed.

PHO has been proposed to be an additional looping factor, as it is preferentially enriched at loop anchors that are maintained upon loss of GAF function [15]. To analyze whether the additional presence of PHO together with GAF is sufficient to mediate PRE looping, we generated a modified construct in which three PHO consensus motifs (GCCCATTT) were inserted into the *Hsp26* promoter sequence (Fig. 4A, Hsp26 + 3xPHO). qChIP analysis confirmed that PHO was successfully recruited to the Hsp26 + 3xPHO construct, while GAF binding levels remained comparable to the unmodified Hsp26 construct (Fig. 4B). Importantly, despite the recruitment of both GAF and PHO, PH was still not detected at the Hsp26 + 3XPho construct (Fig. 4B), and H3K27me3 levels remained low across the *dac* domain (Supplementary Fig. S8B). This suggests that the simultaneous presence of GAF and PHO is not sufficient to recruit PcG proteins and establish a repressive chromatin environment at the *dac* gene locus. To evaluate the functional impact on PRE looping, we performed Hi-C experiments in the Hsp26 + 3xPHO replacement line and quantified the interactions between PRE1 and PRE2 regions (Fig. 4C and D). We observed that the PRE interaction frequencies in the Hsp26 + 3xPHO line were significantly decreased compared to the WT, similar to the Hsp26 line (Fig. 4D).

These results indicate that even the combined presence of GAF and PHO is not sufficient to mediate PRE looping, arguing against the hypothesis that GAF or PHO alone are sufficient to induce looping and instead suggesting that additional factors may be required to establish PRE chromatin contacts.

### The PRE looping specificity is determined by TFs that could form a code

PREs act as binding platforms for a specific combination of TFs that cooperate to recruit PcG complexes and mediate PRE looping. However, only a subset of PREs form loops, and the molecular determinants of PRE–PRE looping specificity remains unclear. The specificity of PRE looping may depend on the quality and the quantity of looping factors occupying the PRE anchors, as well as the specific combination of these factors at the two looping anchors. To test whether a specific TF binding combination encoded in the PRE sequences determine PRE looping specificity, we replaced the *dac* PRE2 with different PRE sequences, namely (i) its orthologous PRE2 sequence from *D. virilis* (*vir*), (ii) a homologous duplicate of the *dac* PRE1 sequence (ect_PRE1), or (iii) unrelated PRE sequences from the *engrailed* (*en*) gene or the Bithorax-Complex (*Fab-7*) loci (Fig. 5A).

We first confirmed that the *vir* PRE recruits GAF and PH when inserted in the *D. melanogaster* genome (Supplementary Fig. S9A). To assess binding at the inserted *Fab-7* and *engrailed* PREs, we used primer pairs spanning the *dac* genomic flank and the inserted PRE sequence, thereby specifically amplifying the right boundary of the insertion (Supplementary Fig. S9B, PRE2 right). We also examined GAF and PH binding at a PRE2-flanking region (D7) and at the neighboring PRE1 (Supplementary Fig. S9C and D). qChIP showed that PH levels at PRE1 are not significantly affected by replacement of PRE2 with unrelated PREs, whereas GAF levels are only moderately reduced, similar to the effect of mutating GAF-binding motifs within PRE2 (Fig. 2B). Both GAF and PH bind to the inserted *Fab-7* and *engrailed* PREs, demonstrating their ability to recruit GAF and Polycomb group (PcG) proteins at the *dac* locus (Supplementary Fig. S9D).

Importantly, all four PRE replacements restored H3K27me3 domain formation and rescued the loss of H3K27me3 caused by PRE2 deletion (Fig. 5A and Supplementary Fig. S9E). Together with the high endogenous GAF and PH occupancy at these elements [9, 43] this indicates that the ectopically inserted sequences retain functional PRE activity and efficiently recruit TFs and PcG proteins. To analyze their ability to mediate PRE looping, we performed Hi-C experiments in the different PRE replacement lines (Fig. 5B–E). As expected, ect_PRE1 partially rescued looping caused by PRE2 deletion, although the looping frequency was slightly reduced compared to WT (Fig. 5C). Interestingly, the orthologous *vir* PRE also rescued looping (Fig. 5C), consistent with the functional conservation of diverse chromatin structural elements between these species [21]. In contrast, the unrelated *Fab-7* PRE, which has been shown to be able to mediate long range PRE interactions between two homologous sequences [53], showed a significantly reduced ability to loop with the *dac* PRE1 compared to the WT or the homologous and orthologous PRE sequences, with similar levels to those of the PRE2 deletion (Fig. 5D and E). Similarly, the *en* PRE replacement showed reduced looping frequency with the PRE1 from the *dac* locus and, although it exhibited partial recovery compared with the PRE2 deletion, this remained significantly lower than the orthologous and homologous PRE interactions (Fig. 5D and E). Notably, the reduction in PRE looping frequency tightly correlates with the penetrance of the previously observed *dac* gain-of-function phenotype [13], emphasizing the functional importance of this PRE loop (Supplementary Fig. S9G). Importantly, at its endogenous locus the *en* PRE forms a chromatin loop with the PRE of the neighboring *invected* gene, demonstrating that this PRE is intrinsically competent to mediate specific PRE interactions. This argues against the idea that looping specificity is determined solely by the binding of looping factors at any given PRE. Instead, they are consistent with the idea that combinations of specific TFs at each PRE may contribute to the specificity of PRE looping interactions.

The *dac* PRE sequence has diverged considerably between *D. melanogaster* and *D. virilis*, sharing only 47% overall identity with short regions of local similarity that mainly correspond to GAF and other motifs (Supplementary Fig. S10A–D). To identify factors that might contribute to *dac* PRE looping specificity, we performed differential *de novo* motif analysis on orthologous *dac* PRE2 sequences relative to *en* and *Fab-7* PREs across diverse *Drosophila* species. Among the top five enriched motifs, four showed no similarity to known TF binding motifs, whereas one corresponded to CA repeats matching the motif of the DNA-binding protein Combgap (Supplementary Fig. S10C–E). Notably, this motif is also present in *dac* PRE1 but absent from the *D. melanogaster en* and *Fab-7* PRE fragments used in our replacement experiments. Given that Combgap is a Polycomb recruitment factor [54] and has recently been implicated in long-range chromatin interactions [55], it represents a strong candidate for future investigation as a potential regulator of PRE looping specificity.

## Discussion

Several lines of evidence suggested a key role of GAF, either alone or in combination with PH, in mediating chromatin looping. However, due to its implication in PcG recruitment, it was so far impossible to disentangle the importance of GAF and PH for PRE looping. In this study, we investigated the direct contribution of GAF in mediating PRE loops and clarified the importance and interplay of GAF and PH for PRE looping. Furthermore, our results provide initial experimental evidence that the binding of specific combinations of compatible TFs at PREs may contribute to the specificity of PRE loops.

Our main findings are summarized below: (i) A tight positive correlation throughout development and across different tissues between GAF binding levels, GAGA motif content and the ability of GAF-bound sites to mediate chromatin loops. Importantly, this correlation was demonstrated both at the genome-wide level and at a specific gene locus (*dac*). (ii) *de-novo* binding of GAF and PH during development is a good predictor for the gain of chromatin looping. (iii) However, PH is dispensable for PRE looping. (iv) Although necessary, GAF is not sufficient to mediate chromatin loops, but requires the presence of additional looping factors, others than PH and PHO. (v) Replacement of a PRE with a homologous and/or orthologous PRE sequence can rescue PRE looping, whereas unrelated PRE sequences are significantly less effective, suggesting that PRE looping specificity is provided by two anchor sequences that recruit a specific set of compatible looping factors.

### The role of GAF in PRE looping

The implication of GAF in 3D chromatin organization has been recognized for over two decades: GAF contains a BTB/POZ oligomerization domain [17–19] that enables long-range enhancer-promoter communication and gene regulation [20, 56]. More recent studies have positioned GAF as a central factor in PRE looping: (i) GAF is enriched at the loop anchors of CREs including TEs [4] and PREs [12], which constitute a specific form of TE loops [13]. (ii) GAF binding is necessary for both intrachromosomal PRE looping at the *dac* gene locus [13] and interchromosomal PRE interactions between homologous copies of the *Fab-7* PREs [24]. (iii) Mutation of GAF affects homologous PRE pairing and deletion of the POZ domain results in the loss of a subset of chromatin loops, particularly those connecting paralogous genes [15].

Here, we clarify the specific role of GAF in PRE looping. Although, *de-novo* binding of GAF during development is predictive for increased chromatin looping, our results demonstrate that GAF binding is necessary but not sufficient for loop formation. Indeed, not all loop anchors are bound by GAF, and a considerable fraction of loops involve GAF at only one anchor (Supplementary Fig. S2C). This asymmetry strongly suggests that additional factors must occupy the opposite anchor to stabilize the interaction, reinforcing the idea that GAF acts in concert with other proteins rather than alone. Importantly, the loss of chromatin looping upon mutation of GAF or its binding sites likely reflects not only the loss of GAF function, but also the concomitant loss of other looping factors that either bind to the same motifs or require GAF for chromatin binding. Therefore, the previously reported essential role of GAF in chromatin looping cannot be attributed to GAF action alone, but rather reflects its functional interaction with additional looping factors that might be equally important to mediate chromatin loops.

### The role of PcG proteins and associated histone marks in PRE looping

Extensive research has linked PcG proteins, particularly PRC1 components, to PRE looping. PcG proteins are known organizers of the 3D genome architecture [28], and PRC1 is critical for both the condensation of Polycomb domains and their long-range interactions in mammals (reviewed in [6]). Moreover, PRC1 has been proposed to act as a looping factor in mammalian cells [57], potentially through oligomerization of the SAM domain of the PRC1 subunit PH. This domain is required for the condensation of Polycomb domains [25] as well as for mediating long-range interactions between them [26, 27]. In line with this, we observed that PH is essential for the compartmentalization of Polycomb domains during *Drosophila* development. Surprisingly however our results show that, although co-gain of PH and GAF binding during development is predictive of PRE looping, genome-wide loss of PH does not disrupt PRE looping. This rules out an essential role for PH in PRE loop formation and instead suggests that PH is passively recruited to PREs by GAF, without contributing directly to loop formation.

A recent study reported that changes in histone modifications, such as H3K27ac, between loop anchors/boundaries are predictive of changes in CTCF/RAD21 loops [58]. Here, we found that H3K27me3, the hallmark of PcG-mediated gene silencing, does not play a major role in PRE looping, since this mark is erased upon loss of PH-binding [30], yet PRE looping is unaffected. We also ruled out the importance of other PRC1 components, such as dRING and PC, for PRE looping. Indeed, upon PH knock down dRING-mediated H2AK118ub is erased from chromatin and PC binding is strongly reduced [30], indicating that the whole PRC1 activity and function is lost.

Together, these findings suggest that PcG proteins and their associated histone modifications are not essential for the formation of PRE loops in *Drosophila*. Instead, the roles of PRC1/PH and H3K27me3 appear to be restricted to higher-order chromatin organization, such as long-range interactions between Polycomb domains. In contrast, PRE loops are essentially mediated by looping factors directly present at loop anchors operating independently of PcG proteins. This distinction allows us to separate intra-domain Polycomb-associated loops, which do not require PcG proteins or their associated histone marks, from long-range inter-domain Polycomb compartment interactions, which depend on PcG components. Notably, in mammals the situation appears different: PRC1 and PRC2 recruitment is largely driven by unmethylated CpG islands rather than discrete TF binding sites [59, 60] and PRC1 is required for both local chromatin compaction and long-range interactions between Polycomb-bound regions [25, 26, 28]. Thus, while PcG proteins contribute primarily to higher-order chromatin architecture in *Drosophila*, in vertebrates they play a more direct role in organizing both short- and long-range chromatin contacts.

### What might be the additional looping factors at PREs?

PREs are built up of a flexible array of DNA binding sites for a multitude of sequence-specific TFs that cooperate to recruit PcG proteins. We showed that mutating increasing numbers of (GA)n repeat motifs result in a gradual loss of GAF and PH binding levels, which negatively correlate with PRE looping efficiency. However, our subsequent finding that PH is not essential and that GAF is not sufficient for PRE looping strongly suggest that other PRE-bound proteins are involved in PRE looping, either via interaction with GAF or by directly binding to (GA)n motifs.

One candidate is PHO, which binds cooperatively with GAF at PREs [61] and is enriched at loop anchors that form independently of GAF [15]. This suggests that PHO might serve as a looping factor at a subset of PREs. However, additional binding of PHO to the GAF-bound Hsp26 sequence does not restore looping, and PHO binding levels are not significantly reduced at the (GA)n_mut2 and (GA)n_mut4 PREs, although PRE looping interactions are significantly decreased. While these observations do not exclude that PHO might play a role in PRE looping, they suggest that other factors must be important.

Pipsqueak (PSQ) is another candidate, as it binds to (GA)n motifs at PRE sequences, interacts with GAF [62] and possesses a BTB-POZ domain that is able to oligomerize. In addition, a specific isoform of PSQ has been implicated in chromatin looping [63]. However, like PHO, PSQ binding to the (GA)n_mut2 and (GA)n_mut4 PREs is not reduced, again indicating that loss of looping is not due to reduced PSQ binding, and suggesting instead that additional TFs/looping factors are required.

In addition, our *de novo* motif analysis identified several enriched sequence motifs at *dac* PREs that do not match any known TF binding sites, suggesting the possible involvement of as-yet-uncharacterized DNA-binding proteins in PRE looping. Notably, the identification of a Combgap-binding motif specifically enriched at *dac* PREs, together with the established role of Combgap in Polycomb recruitment [54] and long-range chromatin interactions [55] highlights Combgap as a strong candidate contributing to PRE looping specificity.

Other proteins that may contribute include CLAMP, a (GA)n-binding protein implicated in long-range chromatin interactions [64], as well as other BTB/POZ domain-containing proteins that interact with GAF, such as LOLAL [65] or BAB1/2 [66], or the recently characterized Vostok protein, which functionally cooperates with GAF in forming a subset of loops in the *Drosophila* brain [67].

In summary, our findings argue that, similar to PcG recruitment to PREs, which involves the combination of many TFs, PRE looping is not governed by a single chromatin factor, but rather depends on the specific combinatorial and quantitative interplay of multiple looping factors at the loop anchors.

### Specificity of PRE loops

The observation that only a subset of PREs form loops raises a fundamental question: what determines the specificity to these chromatin interactions? Importantly, the transcriptional state of the underlying genes does not appear to be a defining factor, as PRE loops occur independently of whether the associated locus is active or repressed [13, 14]. Conversely, several lines of evidence suggest that the sequence content of PREs plays a major role. For instance, sequence homology between PREs has been shown to be important for long-range PRE interactions [53], and many genes associated with PRE loop anchors are paralogous—derived from gene duplications—or are functionally related [15, 16].

However, not all looping interactions can be explained by sequence homology alone. The PRE loop at the *dac* locus, for example, occurs between two nonhomologous sequences associated with unrelated genes (*dac* and *CG5888*). Moreover, the *D. virilis* ortholog of PRE2 can robustly rescue PRE2 function and restore looping with PRE1 at the *dac* locus, although no significant sequence similarity is found when blasting the two orthologous sequences. Finally, prior work has shown that while Polycomb domains, PRC1 binding and TF binding are a well conserved feature of the genome across *Drosophila* species, the underlying PRE DNA sequences evolve rapidly [43]. These findings suggest that it is not primary sequence similarity *per se*, but rather a specific combinatorial TF binding pattern that could form a code in order to ensure PRE compatibility.

Thus, the specificity of PRE loops can be explained by two mutually nonexclusive hypotheses: first, looping may depend on reaching a threshold number of looping factors bound at both PRE anchors, such that PREs capable of looping have accumulated sufficient total binding. Supporting this idea, we observed a positive correlation between GAF binding strength and PRE looping efficiency. Additionally, PREs might contain different combinations of binding sites for TFs, in particular those containing POZ domains [68], and those with the closest match interact with one another to form a specific regulatory loop. Our PRE replacement results are in favor of this hypothesis, as replacing a PRE with a homologous sequence is significantly more efficient in mediating looping interactions compared to unrelated PRE sequences, even when they all recruit high levels of looping factors. This is consistent with the idea that combinations of TFs may help guide PRE–PRE recognition and chromatin looping.

In conclusion, our study clarifies the importance of GAF in PRE looping and reveals that, contrary to prior assumptions, PH is dispensable for local PRE loops. Our findings are consistent with the idea that combinations of TFs may contribute to PRE loop specificity. Given the fact that replacing the *dac* PRE2 with heterologous PREs generally does not fully rescue the *dac* phenotype observed upon deletion of *dac* PRE2, we hypothesize that PRE specificity does play an important regulatory role in the genome, which might in part depend on their ability to set up higher-order 3D chromatin interactions. Future work will be required to decode this combinatorial molecular logic governing PRE looping specificity.

## Supplementary Material

gkag512_Supplemental_Files

## Data Availability

All sequencing data are available at GEO under the series: GSE247377 (CUT&RUN); GSE310299 (Micro-C); GSE310296 (Hi-C). All original codes were deposited on GitHub (https://github.com/cavallifly/Sabaris_et_al_2025) and Zenodo at https://zenodo.org/records/20037311 and are publicly available as of the date of publication. Any additional information required to reanalyze the data reported in this paper is available from the corresponding author upon request. Fly lines are available on request.
