# Supplementary material for: Looping specificity of Polycomb response elements requires GAF and a combination of looping factors that could form a code
Source: Nucleic Acids Res. 2026 May 28;54(10):gkag512. doi: 10.1093/nar/gkag512 (PMC13216745; doi:10.1093/nar/gkag512)
Supplement: gkag512_Supplemental_Files [file gkag512_supplemental_files.zip › 180526140425_NAR_Supplementary_Figures_Sabaris.pdf]

# Supplementary Figure S1

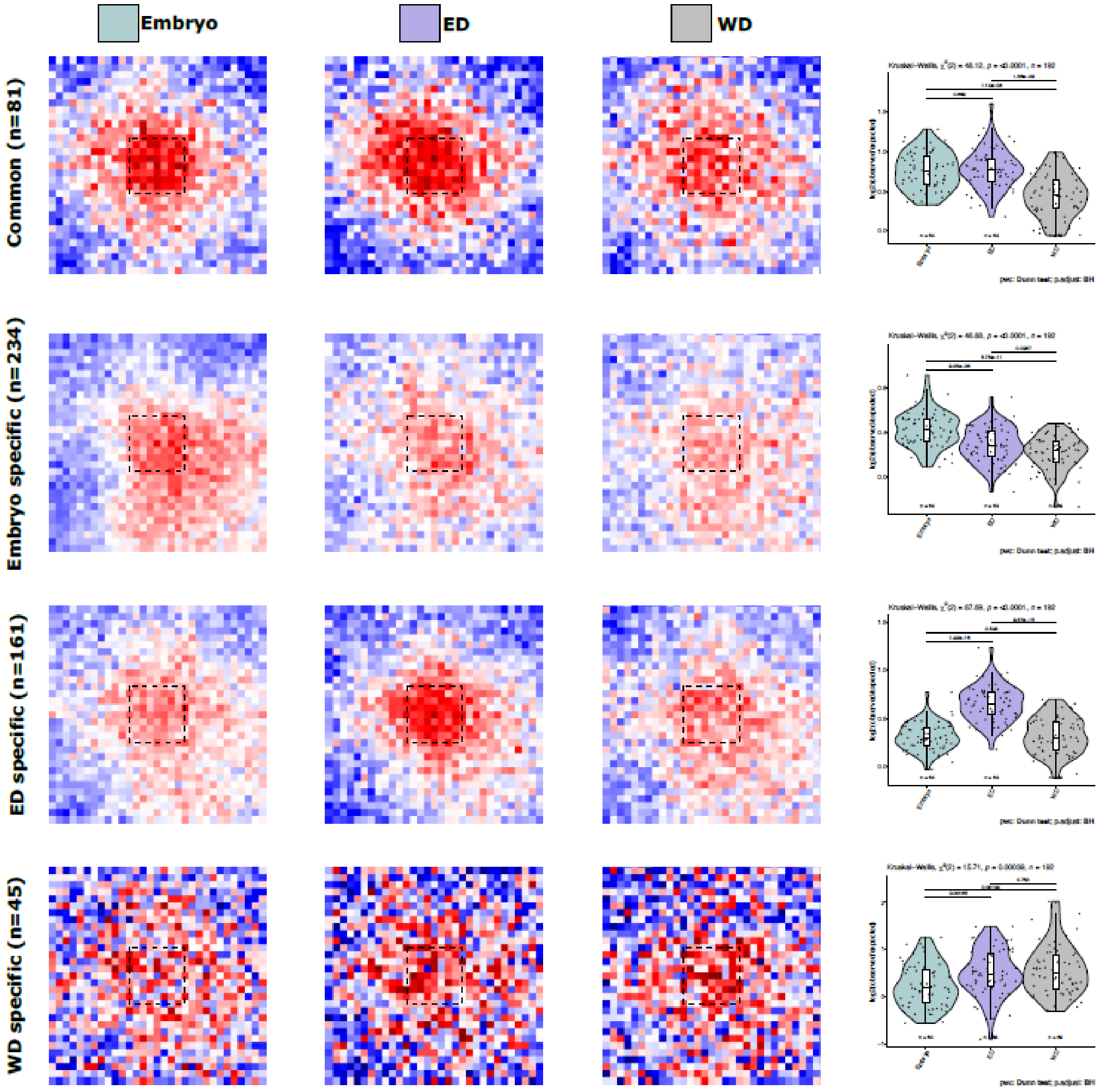


**Supplementary Fig. S1: Stability of chromatin looping during *Drosophila* development**
Aggregate Micro-C map at 250 bp resolutions centered at loops called in all developmental stages (common, Row 1) or condition-specific loops (*Rows 2-4*). Loops are called in embryos, eye imaginal discs (ED) and wing imaginal discs (WD) (see **Supplementary Table S1** and **Material and Methods**).
Violin plot on the right shows the quantification of the signal in the central 8x8 square of the aggregate Micro-C maps. Statistical comparisons were performed using the Kruskal–Wallis test followed by Dunn's post-hoc pairwise test with Benjamini–Hochberg correction. Boxplots show median (central line), Q1=25th and Q3=75th percentiles (box limits), and Q1+1.5×IQR to Q3+1.5×IQR (whiskers), where IQR is the interquartile range.

# Supplementary Figure S2

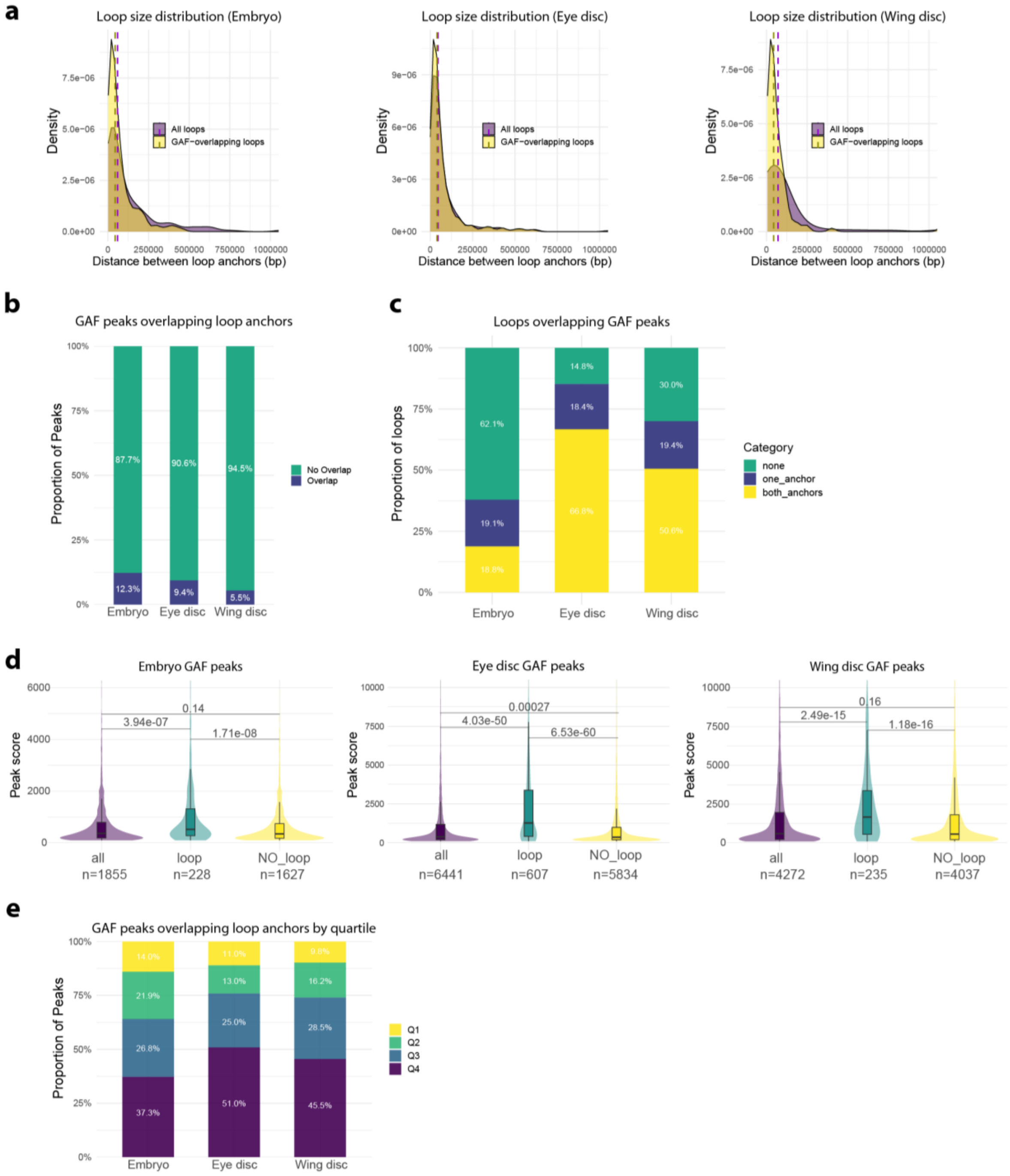

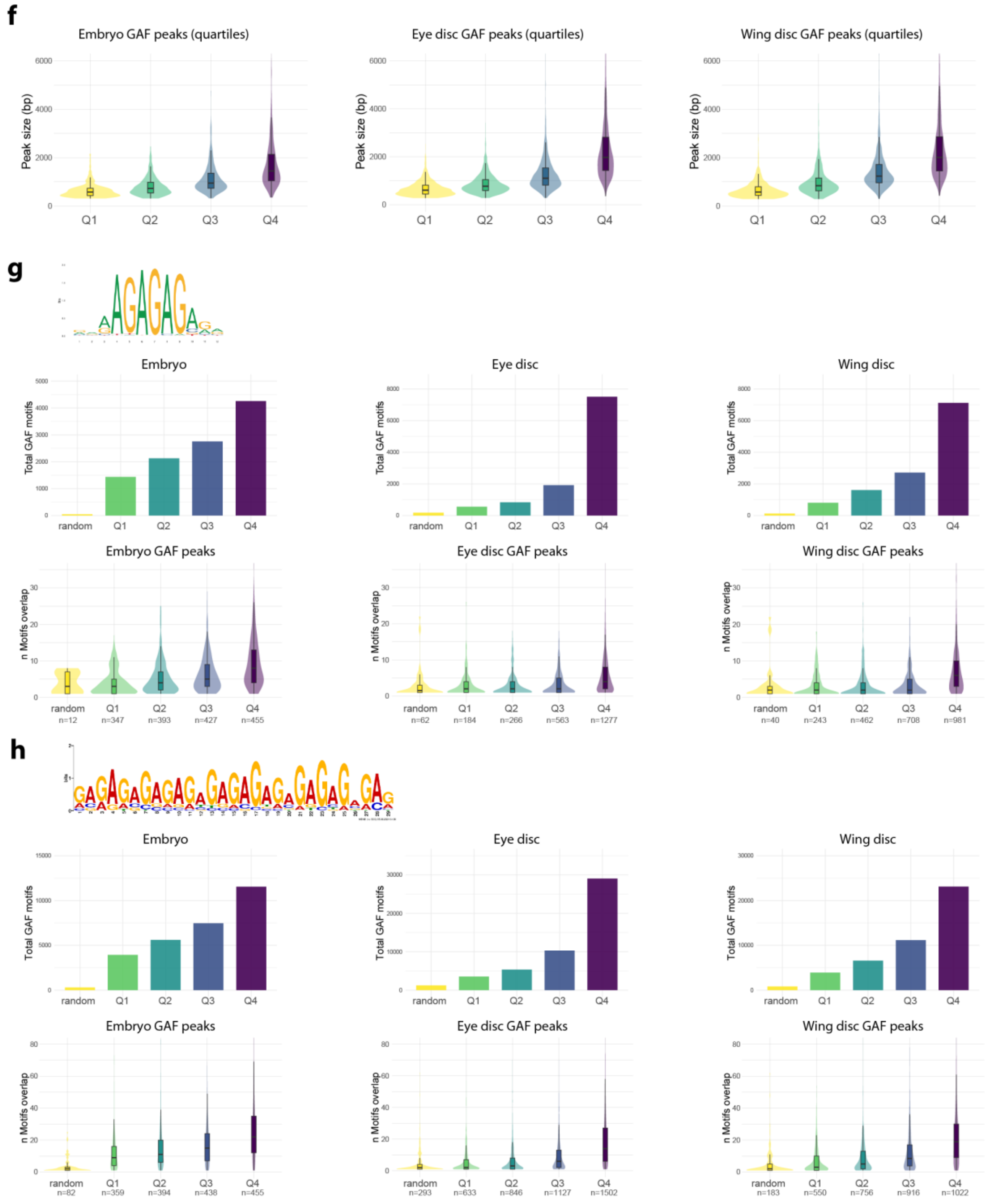


**Supplementary Fig. S2: Characterization of GAF peaks that form chromatin looping**
**(a)** Histograms showing the distribution of distances between loop anchors for all loops and for GAF-overlapping loops (at least one anchor bound by GAF) in embryos and larval eye or wing imaginal discs. **(b)** Percentages of GAF peaks that overlap with loop anchors in embryos and larval eye or wing imaginal discs. **(c)** Percentages of loops that either overlap, in at least one anchor or both anchors, or do not overlap with GAF peaks in embryos and larval eye or wing imaginal discs. **(d)** Violin plots showing MACS3 peak score distributions for all GAF peaks (all), in peaks that overlap with loop anchors (loop) and in peaks that do not loop (NO_loop), in embryos and larval eye or wing imaginal discs. Statistical significance of median differences was assessed using Dunn's test with Benjamini-Hochberg correction. **(e)** Proportion of GAF peaks overlapping with loop anchors, ranked by MACS3 peak score quartiles in embryos and larval eye or wing imaginal discs. **(f)** Violin plots showing the distribution of peak sizes across GAF peak quartiles in embryos and larval eye or wing imaginal discs**. (g-h)** Total number (top) and number of motif-matches per peak (bottom) using either the canonical GAF motif (MA0205.2) **(g)** or the GA29 MEME-1 motif **(h)** in random peaks and GAF peak quartiles from embryos and larval eye or wing imaginal discs.

# Supplementary Figure S3

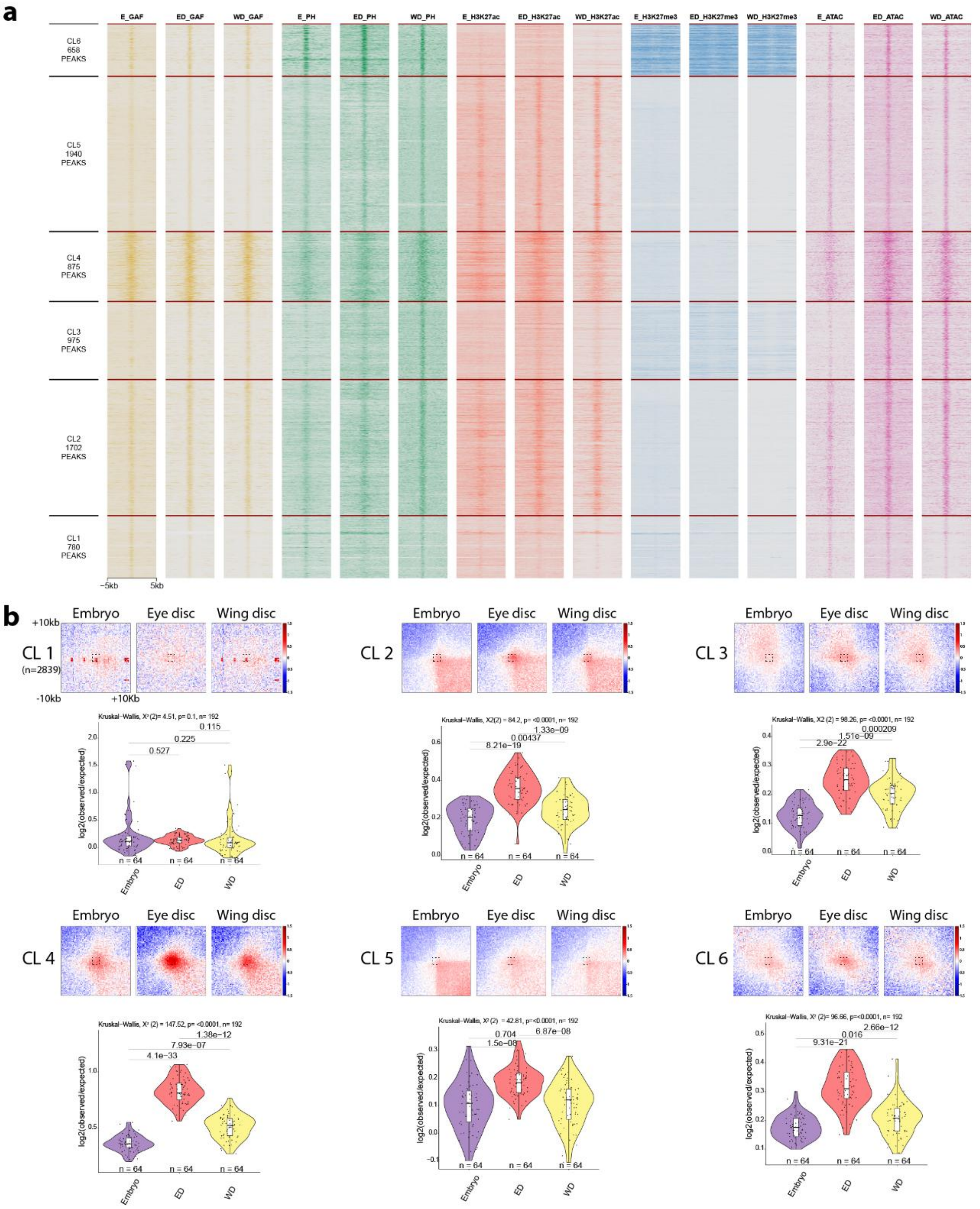


**Supplementary Fig. S3: GAF binding dynamics correlate with chromatin looping**

**(a)** Heatmap showing the *k*-means clustering of GAF, PH, H3K27ac, H3K27me3, and ATAC-seq coverage around all merged GAF peaks from embryos and larval eye and wing imaginal discs (E: embryo; ED: eye disc; WD: wing disc). **(b)** Aggregate Micro-C map in embryos and larval eye and wing imaginal discs at 250 bp resolution centered at GAF peaks clustered as in panel **(a)** at distance separation between 10 and 500 kb and quantification of the signal in the central 8x8 square. Statistical comparisons were performed using the Kruskal–Wallis test followed by Dunn's post-hoc pairwise test with Benjamini–Hochberg correction. Boxplots show median (central line), Q1=25th and Q3=75th percentiles (box limits), and Q1+1.5×IQR to Q3+1.5×IQR (whiskers), where IQR is the interquartile range.

# Supplementary Figure S4

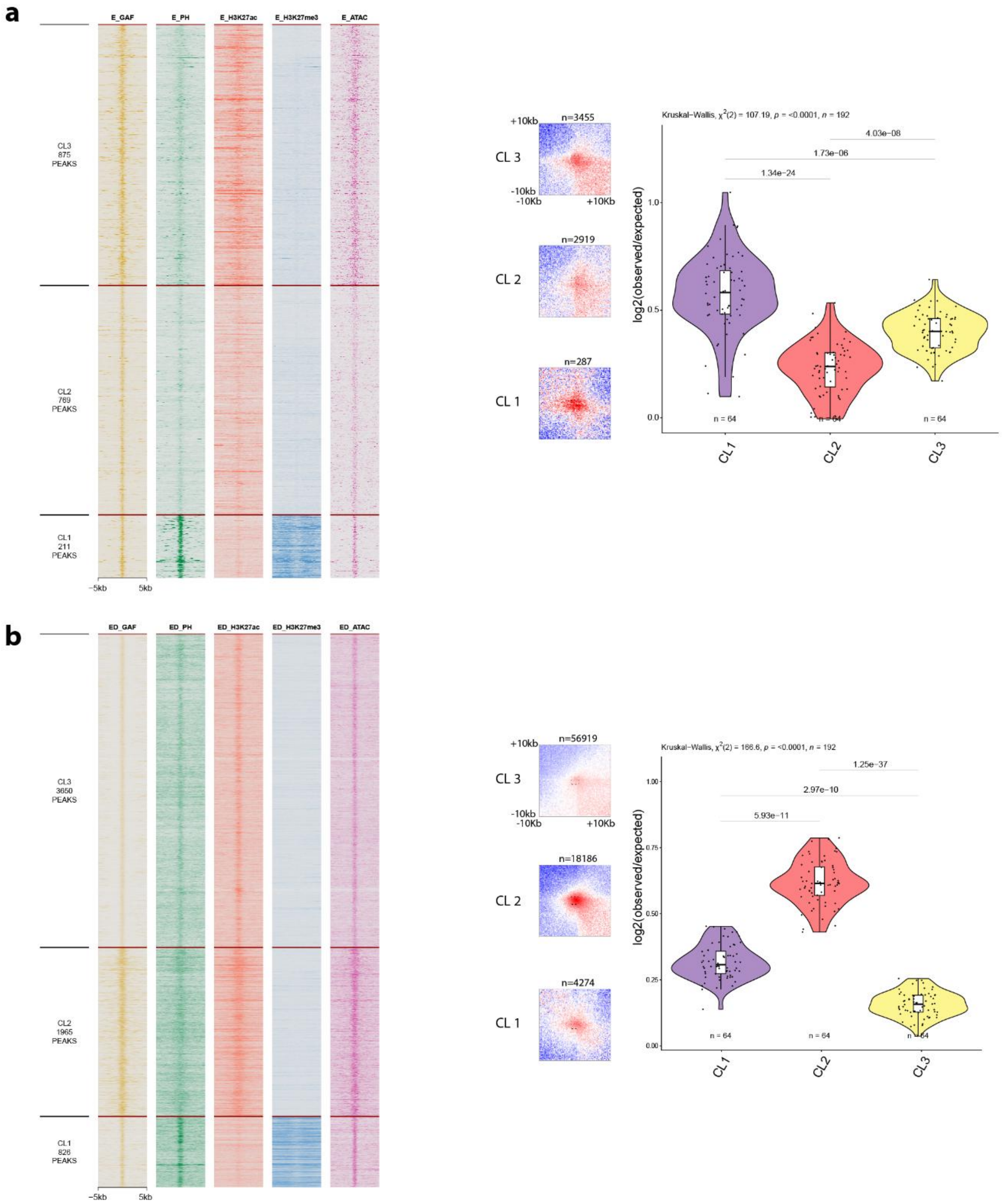

**c**

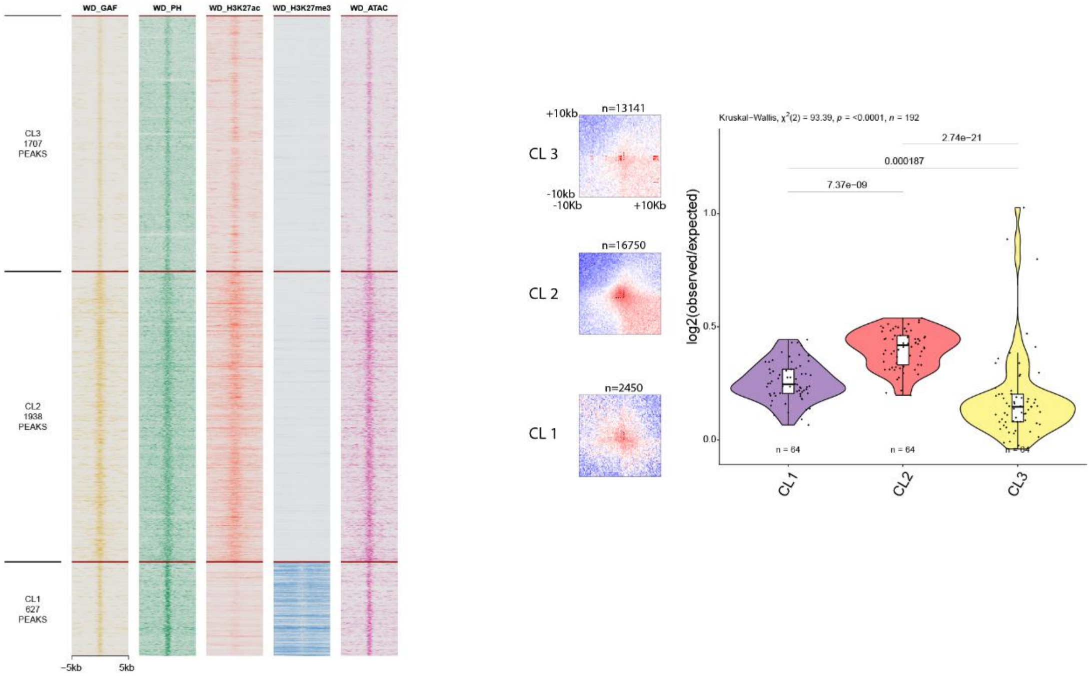


**Supplementary Fig. S4: PREs bound by GAF form strong loop interactions**

**(a-c)** Heatmaps showing *k*-means clustering of GAF, PH, H3K27ac, H3K27me3, and ATAC-seq coverage around GAF peaks in embryos **(a)** and larval eye **(b)** or wing imaginal discs **(c)**. Aggregate Micro-C map at 250 bp resolution centered GAF peaks, which are clustered as in the corresponding left panels at distance separation between 10 and 500 kb and quantification of the signal in the central 8x8 square. Statistical comparisons were performed using the Kruskal–Wallis test followed by Dunn's post-hoc pairwise test with Benjamini–Hochberg correction. Boxplots show median (central line), Q1=25th and Q3=75th percentiles (box limits), and Q1+1.5×IQR to Q3+1.5×IQR (whiskers), where IQR is the interquartile range.

## Supplementary Figure S5

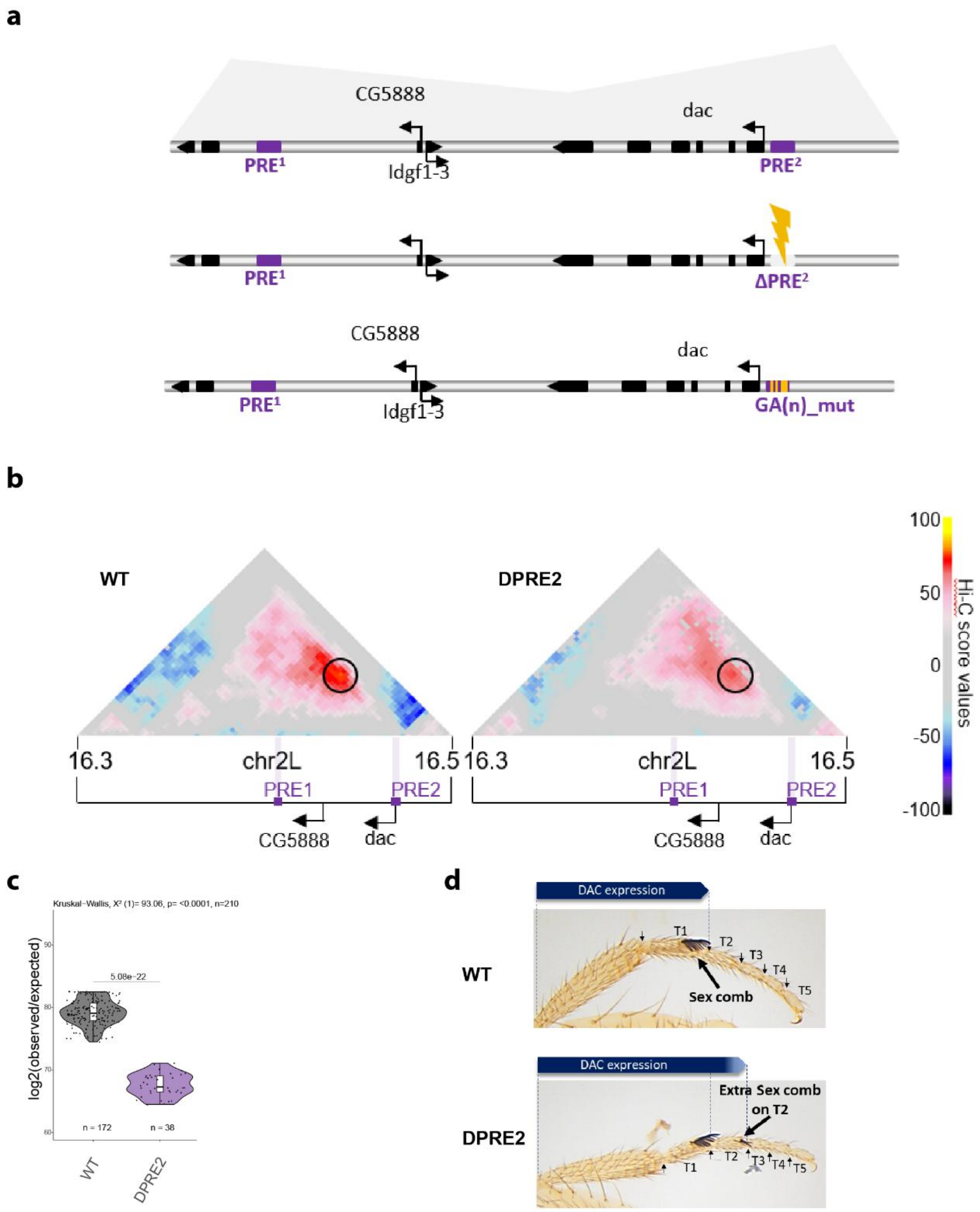


**Supplementary Fig. S5: Mutation of PRE2 sequence results in loss of PRE looping**
**(a)** Schematic representation of the *dac* gene locus and mutated PRE2 lines. **(b)** Hi-C score (**Material and Methods**) maps of a 200 kb region at 3 kb resolution on chromosome 2L at *dac* gene locus in imaginal discs in WT or PRE2 deletion (DPRE2) flies. Black circle indicates the position of the *dac* PRE loop. Violet bars indicate position of PREs. Black arrows indicate promoters of the *dac* and the *CG5888* genes. **(c)** Quantification of the *dac* PRE loop interaction scores. Hi-C interaction score in WT and DPRE2 flies. The number of points per distribution is reported (**Material and Methods**). Boxplots show median (central line), Q1=25th and Q3=75th percentiles (box limits), and Q1+1.5×IQR to Q3+1.5×IQR (whiskers), where IQR is the interquartile range. Statistical comparisons were performed using the Kruskal–Wallis test followed by Dunn's post-hoc pairwise test with Benjamini–Hochberg correction on all the sample considered together as in **Supplementary Fig. S9f**. **(d)** An example of the gain of function phenotype resulting from *dac* overexpression upon PRE2 deletion in the second tarsal segment (Picture adapted from (12)).

## Supplementary Figure S6

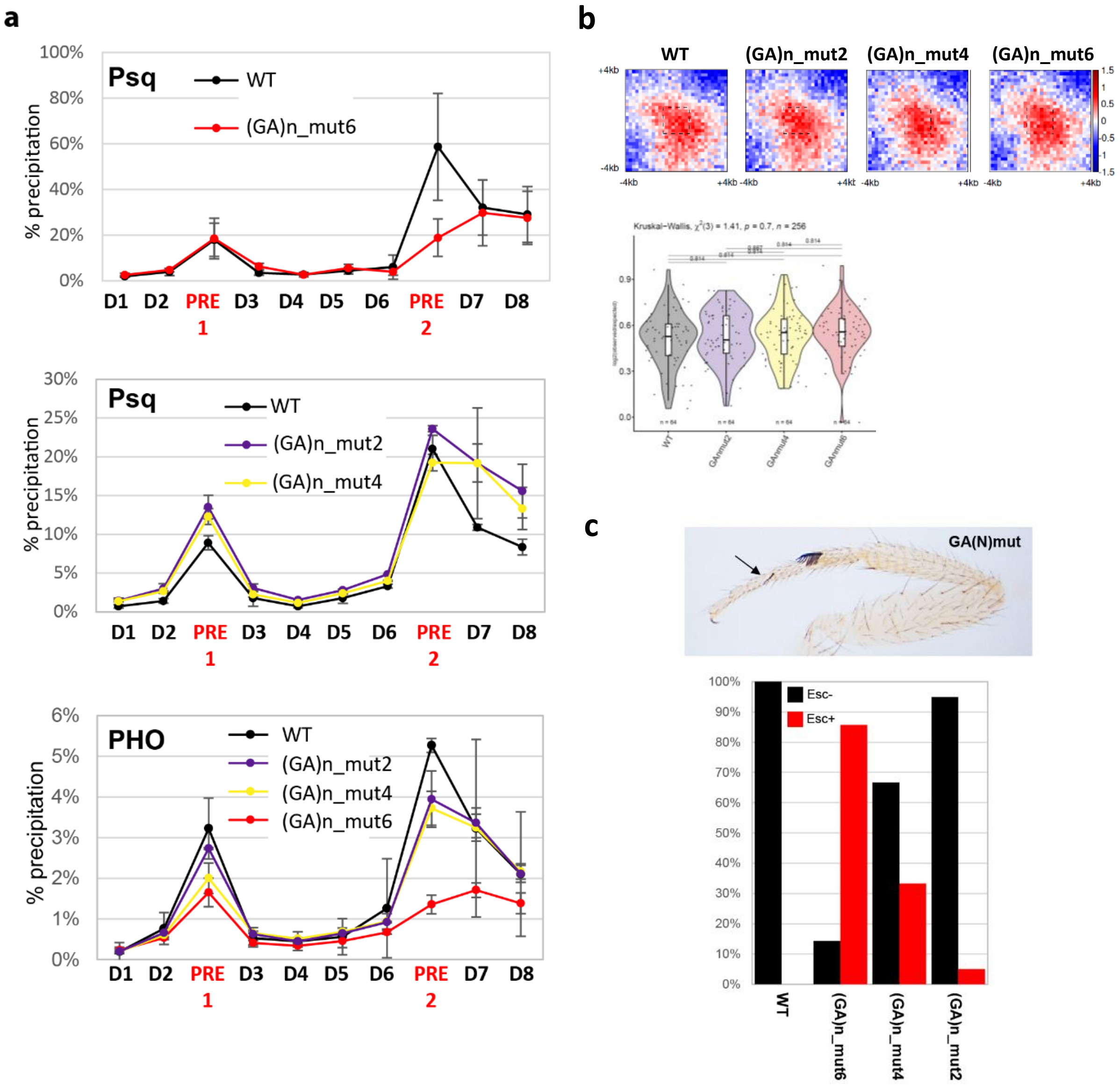


**Supplementary Fig. S6: Consequences of mutating different numbers of GAGA motifs on TF binding**
**(a)** qChIP experiments using PSQ and PHO antibodies in WT, (GA)n_mut2, (GA)n_mut4 and (GA)n_mut6 fly lines. D1-D8, and PRE1, 2 indicate PCR amplicons used for qChIP experiments along the *dac* gene locus (**Supplementary Table S2**). The housekeeping gene *Rp49* was used as negative control, the *engrailed* PRE as a positive control. Data are presented as the mean values ± s.d (error bars) of two independent replicates. **(b)** Aggregate Micro-C map at 250 bp resolutions centered at all loops called in Drosophila embryos (see **Supplementary Table S1**). Violin plot shows the quantification of the signal in the central 8x8 square of the aggregate Micro-C maps. Statistical comparisons were performed using the Kruskal–Wallis test followed by Dunn's post-hoc pairwise test with Benjamini–Hochberg correction. **(c)** Quantification of the extra sex comb (ESC) phenotype in the indicated fly lines grown at 28°C. A minimum of 50 male flies was scored. Above a representative example of a mutant phenotype leg is shown.

# Supplementary Figure S7

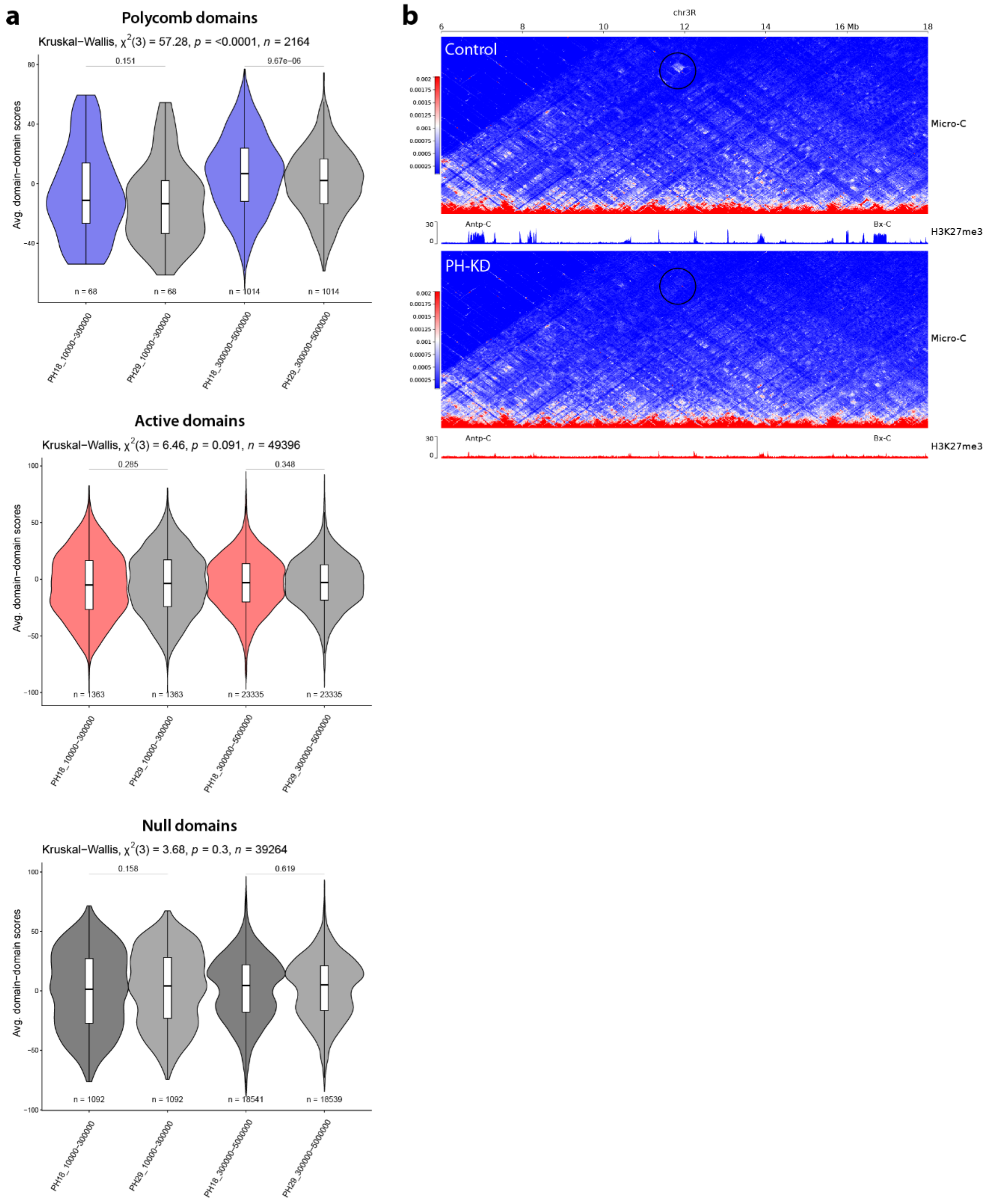


**Supplementary Fig. S7: Loss of PH function specifically affects Polycomb domain interactions.**
**(a)** Violin plot shows the quantification of the average score between PcG (*top*), Active (*middle*), and Null (*bottom*) domains identified using ChromHMM (https://ernstlab.github.io/ChromHMM/) (45) at 20 kb resolution from ATAC-seq, H2AK118Ub, H3K27ac, and H3K27me3 signals in eye disc Control samples. Specifically, PcG domains are enriched in H3K27me3, H2AK118Ub, and ATAC-seq signals, Active domains in H3K27ac and ATAC-seq signals, and Null ones in none of the analyzed marks. Inter-domain scores are classified in shorter (from 10 to 300 kb) and longer-range (from 300 kb to 5 Mb) of genomic separation. Statistical comparisons were performed using the Kruskal–Wallis test followed by Dunn's post-hoc pairwise test with Benjamini–Hochberg correction. Boxplots show median (central line), Q1=25th and Q3=75th percentiles (box limits), and Q1+1.5×IQR to Q3+1.5×IQR (whiskers), where IQR is the interquartile range. **(b)** Micro-C contact maps in control eye discs (top) or upon loss of PH function (PH-KD, bottom) showing the loss of long-range contacts between the Antp-C and the Bx-C (black circle).

# Supplementary Figure S8

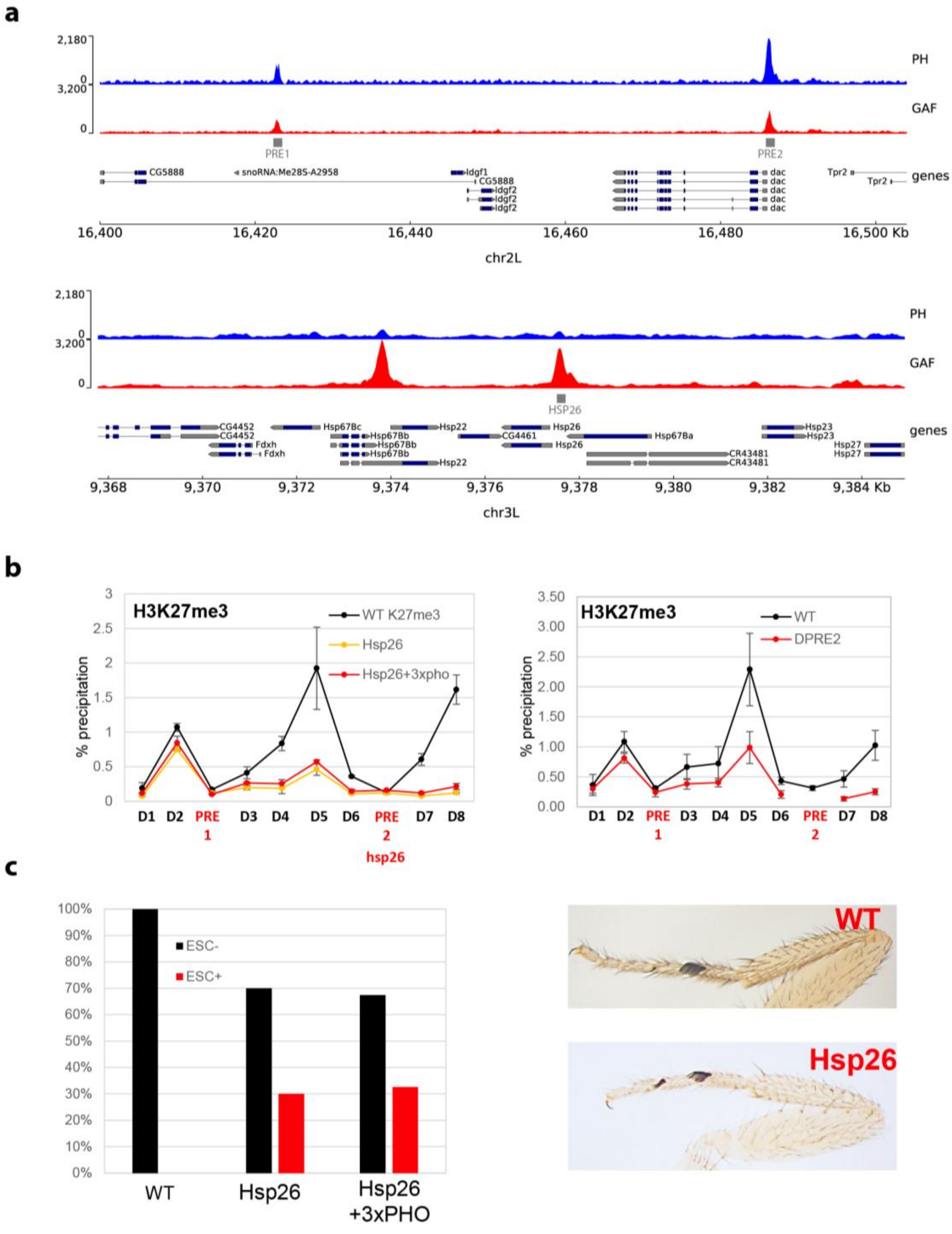


**Supplementary Fig. S8: Hsp26 sequences do not recruit PcG proteins in the context of a PcG domain**
**(**a) CUT&RUN PH and GAF profiles at the *dac* gene locus on chromosome 2L (top), or the *hsp26* gene locus on chromosome 3L (bottom). **(b)** qChIP experiments using H3K27me3 antibody in WT, Hsp26, and Hsp26+3xPHO fly lines (left), or DPRE2 line (right). D1-D8, and PRE1, 2 indicate PCR amplicons used for qChIP experiments along the *dac* gene locus (**Supplementary Table S2**). The housekeeping gene *Rp49* was used as negative control, the *engrailed* PRE as a positive control. Data are presented as the mean values ± s.d (error bars) of two independent replicates. **(c)** Quantification of the extra sex comb (ESC) phenotype in the indicated fly lines grown at 28°C. A minimum of 50 male flies were scored (left). Representative examples of a WT and ESC phenotype leg are shown (right).

# Supplementary Figure S9

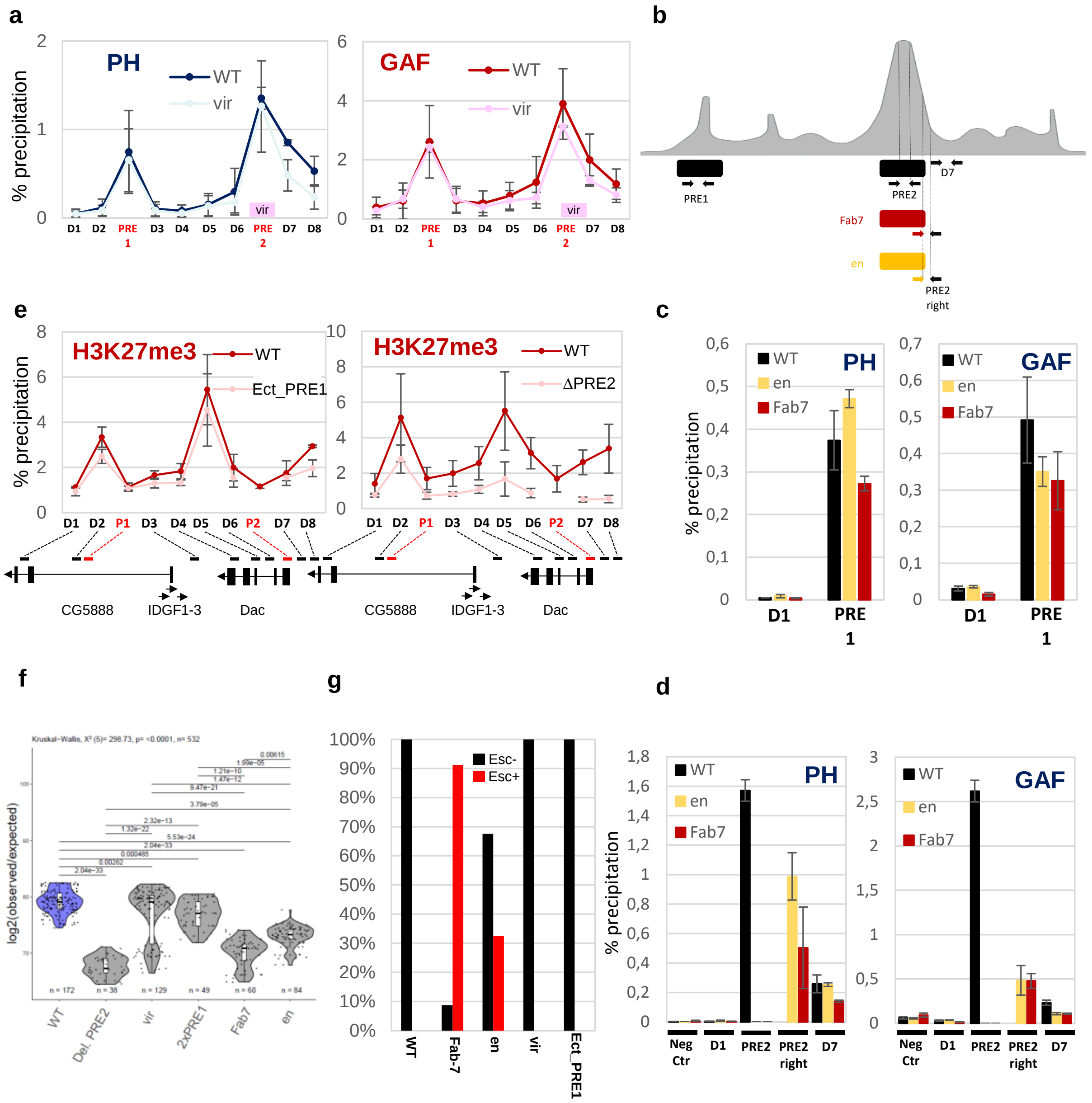

**Supplementary Fig. S9: The orthologous PRE sequence from *D. virilis* recruits GAF and PH proteins in *D. melanogaster***
**(a)** qChIP experiments using PH or GAF antibodies in WT flies or PRE-mutant flies where *dac* PRE2 has been replaced by the orthologous sequence from *D. virilis* (vir). D1-D8, and PRE1, 2 indicate PCR amplicons used for qChIP experiments along the *dac* gene locus in panels **(a)** and **(e)** (**Supplementary Table S2**). The housekeeping gene *Rp49* was used as negative control, the *engrailed* PRE as a positive control. Data are presented as the mean values ± s.d (error bars) of two independent replicates. **(b)** Schematic representation of the location of primer pairs used to study PH and GAF binding to ectopically inserted PREs **(c), (d)** qChIP experiments using PH or GAF antibodies in WT flies or PRE-mutant flies where *dac* PRE2 has been replaced by the *engrailed* (en) PRE or the Fab-7 PRE. Data are presented as the mean values ± s.d (error bars) of two independent replicates.
**(e)** qChIP experiments using H3K27me3 antibody in WT flies or PRE-mutant flies where *dac* PRE2 has been replaced by PRE1 (Ect_PRE1) or deleted (ΔPRE2). **(f)** Hi-C interaction score at the *dac* PRE in WT, ΔPRE2, and mutant PRE lines, where the *dac* PRE2 has been replaced by the indicated PREs (vir, ect_PRE1, Fab-7 and en). The number of points per distribution is reported (**Material and Methods**). Boxplots show median (central line), Q1=25th and Q3=75th percentiles (box limits), and Q1+1.5×IQR to Q3+1.5×IQR (whiskers), where IQR is the interquartile range. Statistical comparisons were performed using the Kruskal–Wallis test followed by Dunn's post-hoc pairwise test with Benjamini–Hochberg correction. **(g)** Quantification of the extra sex comb (ESC) phenotype in the indicated fly lines grown at 28°C. A minimum of 50 male flies were scored.

# Supplementary Figure S10

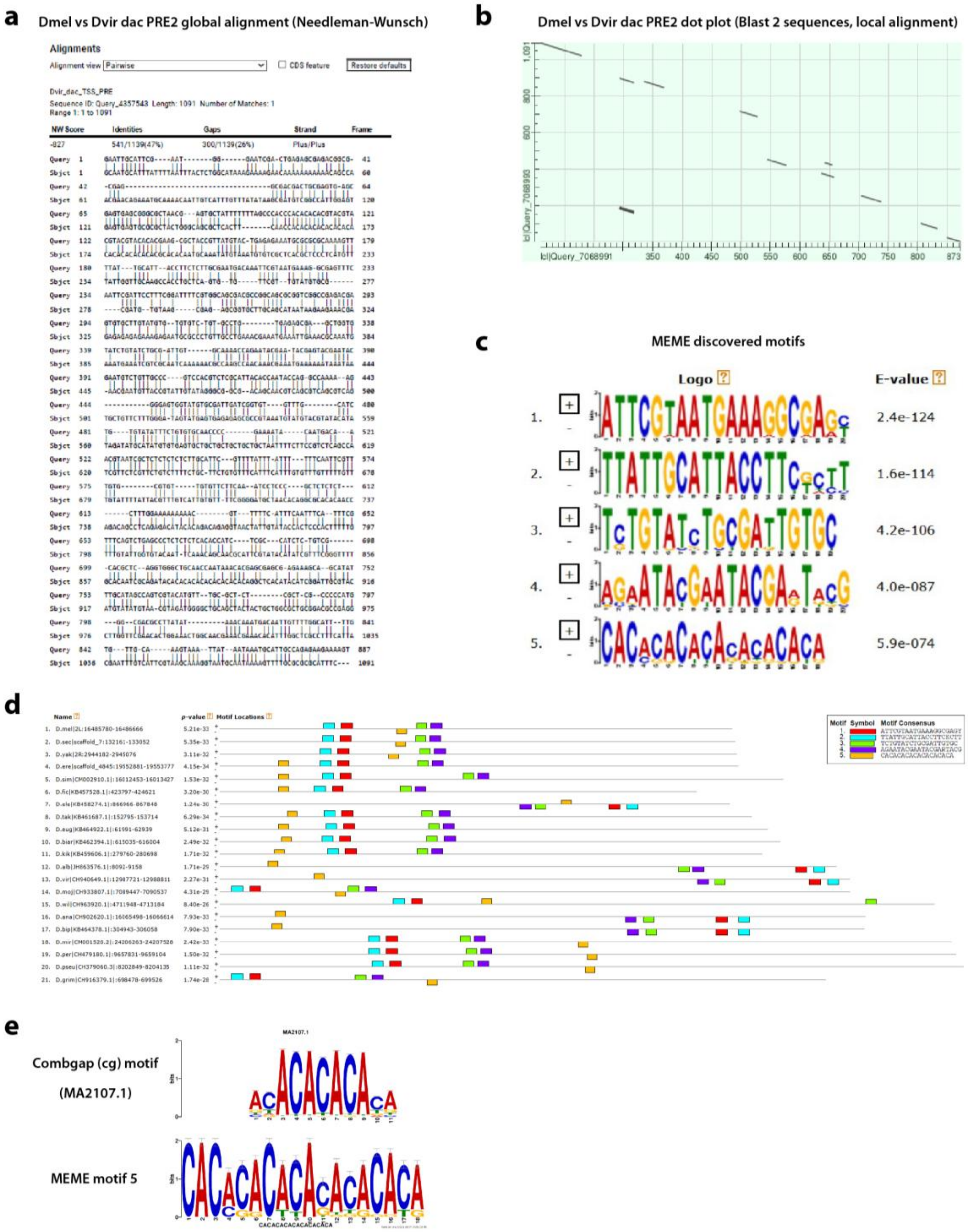


**Supplementary Fig. S10. Comparative sequence analysis and motif discovery for *dac* PRE2 orthologs.** **(a)** Global alignment of *D. melanogaster* and *D. virilis dac* PRE2 sequences performed using the Needleman–Wunsch algorithm. **(b)** Dot plot representation of local sequence homology between *D. melanogaster* and *D. virilis dac* PRE2 sequences generated using BLAST (BLAST 2 sequences). **(c)** Top five motifs identified by *de novo* motif discovery using MEME in discriminative mode, with *dac* PRE2 orthologous sequences from *Drosophila* species as the primary set and *engrailed* (*en*) and Fab-7 PRE homologous sequences as the control set. **(d)** Distribution of the five motifs identified in **(c)** across the 21 *Drosophila* species *dac* PRE2 orthologous sequences analyzed. **(e)** Significant match identified by Tomtom between MEME motif 5 and the JASPAR2026_CORE_insects_non-redundant database, corresponding to the binding site of the DNA-binding protein Combgap (MA2107.1).

**Supplementary Table S1:** Sequencing statistics and processed data of the CUT&RUN, Micro-C and Hi-C samples generated for this work.

**Supplementary Table S2:** Primer sequences used for the generation of fly lines and qCHIP
